# Maternal diabetes and risk of attention-deficit/hyperactivity disorder in offspring in a multinational cohort of 3.6 million mother–child pairs

**DOI:** 10.1038/s41591-024-02917-8

**Published:** 2024-04-08

**Authors:** Adrienne Y. L. Chan, Le Gao, Miyuki Hsing-Chun Hsieh, Lars J. Kjerpeseth, Raquel Avelar, Tobias Banaschewski, Amy Hai Yan Chan, David Coghill, Jacqueline M. Cohen, Mika Gissler, Jeff Harrison, Patrick Ip, Øystein Karlstad, Wallis C. Y. Lau, Maarit K. Leinonen, Wing Cheong Leung, Tzu-Chi Liao, Johan Reutfors, Shih-Chieh Shao, Emily Simonoff, Kathryn Choon Beng Tan, Katja Taxis, Andrew Tomlin, Carolyn E. Cesta, Edward Chia-Cheng Lai, Helga Zoega, Kenneth K. C. Man, Ian C. K. Wong

**Affiliations:** 1 Department of Pharmacology and Pharmacy, Li Ka Shing Faculty of Medicine, University of Hong Kong, Hong Kong, Hong Kong; 2 Laboratory of Data Discovery for Health (D24H), Hong Kong Science Park, Pak Shek Kok, Hong Kong; 3 Research Department of Practice and Policy, UCL School of Pharmacy, London, UK; 4 Groningen Research Institute of Pharmacy, Unit of PharmacoTherapy, Epidemiology and Economics, University of Groningen, Groningen, The Netherlands; 5 School of Pharmacy, Institute of Clinical Pharmacy and Pharmaceutical Sciences, College of Medicine, National Cheng Kung University, Tainan, Taiwan; 6 Department of Chronic Diseases, Norwegian Institute of Public Health, Oslo, Norway; 7 Institute of Biological Psychiatry, Mental Health Centre Sct Hans, Mental Health Services, Copenhagen, Denmark; 8 Department of Child and Adolescent Psychiatry and Psychotherapy, Central Institute of Mental Health, Medical Faculty Mannheim, University of Heidelberg, Heidelberg, Germany; 9 School of Pharmacy, Faculty of Medical and Health Sciences, The University of Auckland, Auckland, New Zealand; 10 Departments of Paediatrics and Psychiatry, Faculty of Medicine, Dentistry and Health Sciences, University of Melbourne, Melbourne, Victoria Australia; 11 Murdoch Children’s Research Institute, Melbourne, Victoria Australia; 12 Centre for Fertility and Health, Norwegian Institute of Public Health, Oslo, Norway; 13 Centre for Pharmacoepidemiology, Department of Medicine, Karolinska Institutet, Solna, Sweden; 14 Academic Primary Health Care Centre, Region Stockholm, Stockholm, Sweden; 15 Department of Molecular Medicine and Surgery, Karolinska Institutet, Stockholm, Sweden; 16 Research Centre for Child Psychiatry, University of Turku, Turku, Finland; 17 Department of Paediatrics and Adolescent Medicine, Li Ka Shing Faculty of Medicine, The University of Hong Kong, Hong Kong, Hong Kong; 18 Knowledge Brokers, Finnish Institute for Health and Welfare, Helsinki, Finland; 19 Department of Obstetrics and Gynaecology, Kwong Wah Hospital, Yau Ma Tei, Hong Kong; 20 Department of Pharmacy, Keelung Chang Gung Memorial Hospital, Keelung, Taiwan; 21 Department of Child and Adolescent Psychiatry, King’s College London, Institute of Psychiatry, Psychology and Neuroscience, London, UK; 22 Department of Medicine, School of Clinical Medicine, University of Hong Kong, Hong Kong, Hong Kong; 23 Centre of Public Health Sciences, Faculty of Medicine, University of Iceland, Reykjavik, Iceland; 24 School of Population Health, Faculty of Medicine and Health, University of New South Wales, Sydney, New South Wales Australia; 25 Centre for Medicines Optimisation Research and Education, University College London Hospitals NHS Foundation Trust, London, UK; 26 School of Pharmacy, Medical Sciences Division, Macau University of Science and Technology, Taipa, Macau; 27 Advance Data Analytics for Medical Science Limited, Hong Kong, Hong Kong; 28 School of Pharmacy, Aston University, Birmingham, UK

**Keywords:** Gestational diabetes, ADHD

## Abstract

Previous studies report an association between maternal diabetes mellitus (MDM) and attention-deficit/hyperactivity disorder (ADHD), often overlooking unmeasured confounders such as shared genetics and environmental factors. We therefore conducted a multinational cohort study with linked mother–child pairs data in Hong Kong, New Zealand, Taiwan, Finland, Iceland, Norway and Sweden to evaluate associations between different MDM (any MDM, gestational diabetes mellitus (GDM) and pregestational diabetes mellitus (PGDM)) and ADHD using Cox proportional hazards regression. We included over 3.6 million mother–child pairs between 2001 and 2014 with follow-up until 2020. Children who were born to mothers with any type of diabetes during pregnancy had a higher risk of ADHD than unexposed children (pooled hazard ratio (HR) = 1.16, 95% confidence interval (CI) = 1.08-1.24). Higher risks of ADHD were also observed for both GDM (pooled HR = 1.10, 95% CI = 1.04-1.17) and PGDM (pooled HR = 1.39, 95% CI = 1.25-1.55). However, siblings with discordant exposure to GDM in pregnancy had similar risks of ADHD (pooled HR = 1.05, 95% CI = 0.94-1.17), suggesting potential confounding by unmeasured, shared familial factors. Our findings indicate that there is a small-to-moderate association between MDM and ADHD, whereas the association between GDM and ADHD is unlikely to be causal. This finding contrast with previous studies, which reported substantially higher risk estimates, and underscores the need to reevaluate the precise roles of hyperglycemia and genetic factors in the relationship between MDM and ADHD.

## Abstract (web summary)

In a large multinational cohort study, maternal, gestational or pregestational diabetes was associated with only a small-to-moderate risk of ADHD in offspring, contrary to previous estimates that showed stronger effect sizes, attributing the differences in findings to confounding by shared genetic and familial factors.

## Main

Globally, 16% of pregnant women experience hyperglycemia^1,2^. The prevalence of maternal diabetes mellitus (MDM) has increased worldwide, which is associated with the growing epidemic of obesity, advancing maternal age and improved diagnostic approaches for MDM^3,4^. There are calls for greater attention to the risks associated with diabetes in pregnancy given the increasing trend of gestational diabetes and preexisting type 2 diabetes^5^. Animal studies have demonstrated the adverse effects of hyperglycemia during pregnancy on inflammatory responses, intrauterine oxidative stress and imbalance in epigenetic mechanisms, which may contribute to poor neurodevelopment in the offspring^6,7^.

Attention-deficit/hyperactivity disorder (ADHD) is a neurodevelopmental disorder characterized by hyperactivity, impulsivity and inattentiveness^8^. Currently, ADHD is estimated to affect 2% to 7% of children worldwide^9,10^, making it one of the most common disorders among school-aged children. ADHD not only adversely impacts the affected individuals but also poses a substantial burden on their families and the wider society^11–17^. A complex interaction between genetic, environmental and psychosocial risk factors is thought to be responsible for the etiology of ADHD^8^.

Emerging evidence has suggested that both pregestational diabetes mellitus (PGDM) and gestational diabetes mellitus (GDM) are associated with ADHD. A previous meta-analysis found that the offspring of diabetic mothers were at 40% higher risk of ADHD^18^. However, some of the included studies used self-reported data^19–21^, had limited statistical power^20,22^ or had limited adjustment for confounders^19,20,23,24^, especially familial factors, and were predominantly conducted in White populations^7,19–23,25,26^. To account for these limitations, we conducted the current cohort study based on population-based data covering over 3.6 million mother–child pairs in Hong Kong, New Zealand, Taiwan, Finland, Iceland, Norway and Sweden, with extensive coverage of relevant covariates, to assess the association between MDM and the risk of ADHD in offspring.

## Results

**Extended Data Fig. 1:**
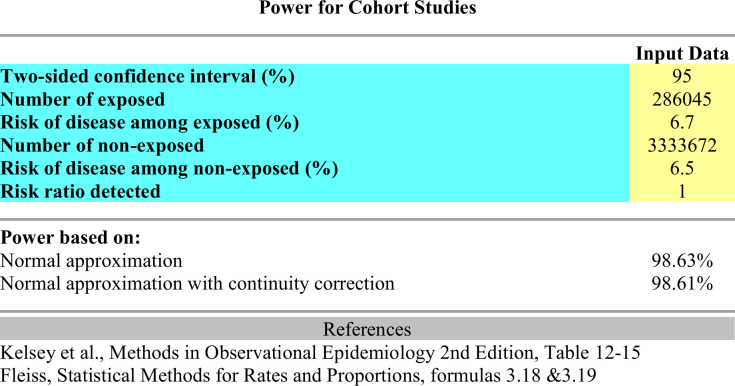
Sample size and power considerations. Notes: Results are rounded up to the nearest integer. Abbreviations: CC, continuity correction.

This study consisted of children from all live births within the site-specific study period (Table 1). All mother–child pairs were linked with exact deterministic linkage^27^. Children without valid mother–child linkage or with incomplete birth information (for example, sex or gestational age) or without at least 6 years of follow-up were excluded to allow sufficient follow-up time to capture ADHD outcomes, as a diagnosis is often deferred until a child is of school age^28^. The follow-up period for each child started on the date of birth and ended on the date of outcome occurrence, date of death, or end of data source-specific study period, whichever came first. Details of the sample size calculation are given in Extended Data Fig. 1.

**Table 1:** Data characteristics

Site	Data source	Nature of source data	Coverage	Time period	Mother–child pairs included, *N*
Hong Kong	Clinical Data Analysis and Reporting System	Electronic health record from public hospital and clinics	Territory-wide (~11 m)	Births 2001-2014, follow-up until 2020	382,338
Finland	NorPreSS	National registers	Nationwide (~5.5 m)	Births 2006-2014, follow-up until 2020	532,004
Iceland	NorPreSS	National registers	Nationwide (~350,000)	Births 2004-2011, follow-up until 2017	32,251
Norway	NorPreSS	National registers	Nationwide (~5.3 m)	Births 2005-2014, follow-up until 2020	575,631
Sweden	NorPreSS	National registers	Nationwide (~10 m)	Births 2006-2013, follow-up until 2019	790,533
New Zealand	Ministry of Health National Data Collection	National registers	Nationwide (~5 m)	Births 2007-2014, follow-up until 2020	520,143
Taiwan	NHIRD	Insurance claims database	Nationwide (~23 m)	Births 2011-2014, follow-up until 2020	789,730

m, million; NHIRD, National Health Research Insurance Database; NorPreSS, Nordic Pregnancy Drug Safety Studies.

**Fig. 1:**
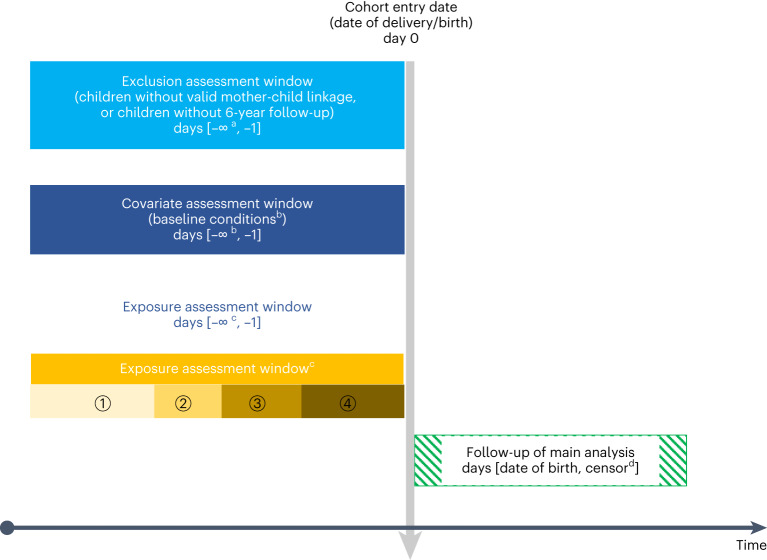
Illustration of cohort inclusion and pregnancy periods. ^a^The earliest date of mothers’ health data in each data source. ^b^Baseline conditions included: demographics, maternal conditions and medication use. For HK analysis, maternal age at delivery and birth year were assessed at the date of delivery, body mass index (BMI) was assessed from LMP − 365 days to LMP − 1 day and all other covariates were assessed before LMP; for analysis in Nordic countries and New Zealand, medication use was assessed from LMP − 365 days to LMP − 1 day, and diagnoses were assessed from LMP − 365 days to delivery date; for Taiwan analysis, all covariates were assessed within 2 years before the date of delivery. ^c^Exposure window: (1) Period before pregnancy, (2) first trimester: LMP to LMP + 90 days, (3) second trimester: LMP + 91 days to LMP + 180 days, and (4) third trimester: LMP + 181 days to delivery date. ^d^Earliest of: date of ADHD diagnosis, date of first ADHD medication prescription, date of death, end of database catchment period.

**Extended Data Fig. 2:**
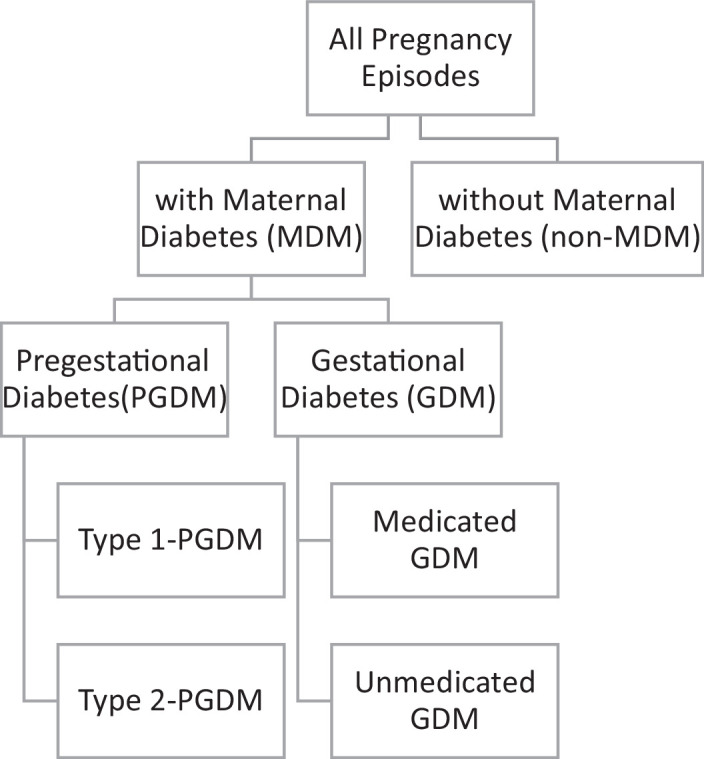
General definition of exposure groups. Abbreviations: GDM, gestational diabetes mellitus; MDM, maternal diabetes mellitus; PGDM, pregestational diabetes mellitus.

We identified the start and end of pregnancy by the date of the last menstrual period (LMP) and the child’s date of birth (Fig. 1). In Hong Kong, New Zealand and the Nordic countries, LMP was determined by subtracting gestational age at birth (determined by ultrasound) from the date of birth; in Taiwan, this was defined as the date of delivery minus 280 days^29^. As hyperglycemia may affect neurodevelopment differently at different trimesters, we divided the pregnancy period into first trimester (0–90 days after the LMP), second trimester (91–180 days after the LMP) and third trimester (181 days after the LMP to delivery). The primary exposure is MDM (including GDM and PGDM). MDM was further classified as GDM (including those receiving and not receiving medications) and PGDM (including type 1 and type 2 PGDM; Extended Data Fig. 2). PGDM refers to existing diabetes before pregnancy, and GDM refers to diabetes diagnosed only during pregnancy. Data source-specific criteria were applied to ensure the best identification of the exposure status (Supplementary Table 1).

ADHD was defined by each site using specific diagnosis and medication codes as reported in previous studies and detailed in Supplementary Table 1 (refs. ^30,31^). In Hong Kong, New Zealand and Taiwan, children with either one ADHD diagnosis or one ADHD medication prescription were regarded as having the outcome. In Nordic countries, the outcome was defined as (1) ≥2 records of ADHD diagnoses or (2) ≥1 record of ADHD diagnosis and ≥2 records of ADHD medication prescription fills. In Hong Kong, New Zealand and Nordic countries, these criteria needed to be met at or after the age of 3 years to exclude invalid ADHD diagnoses (Supplementary Table 1).

**Fig. 2:**
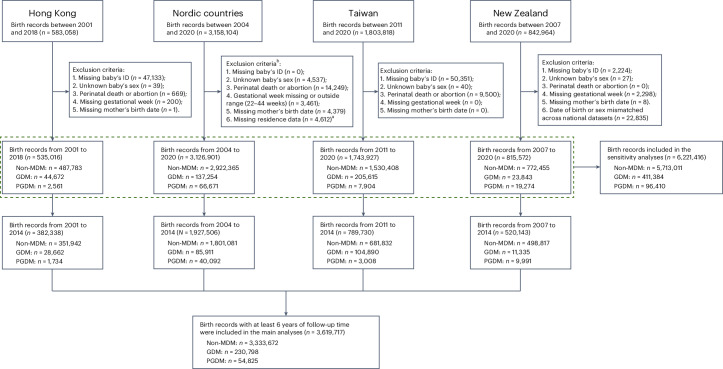
Flowchart of cohort identification. ^a^Not applicable to Finland data, where residence/migration data are not available. ^b^Individuals could fulfill more than one exclusion criteria.

We identified 3,619,717 mother–child pairs to be included in the analysis (Fig. 2). Overall, 8.0% (*n* = 30,396), 4.1% (*n* = 21,326), 13.7% (*n* = 107,898) and 6.6% (*n* = 126,425) of children were born to mothers with diabetes in Hong Kong, New Zealand, Taiwan and the Nordic countries, respectively. Child and maternal characteristics are summarized in Supplementary Table 2. Covariate balances were achieved after propensity score (PS) weighting with standardized differences <10%, except for obesity in New Zealand (Supplementary Table 3).

### Primary analyses and sibling-matched comparisons

**Fig. 3:**
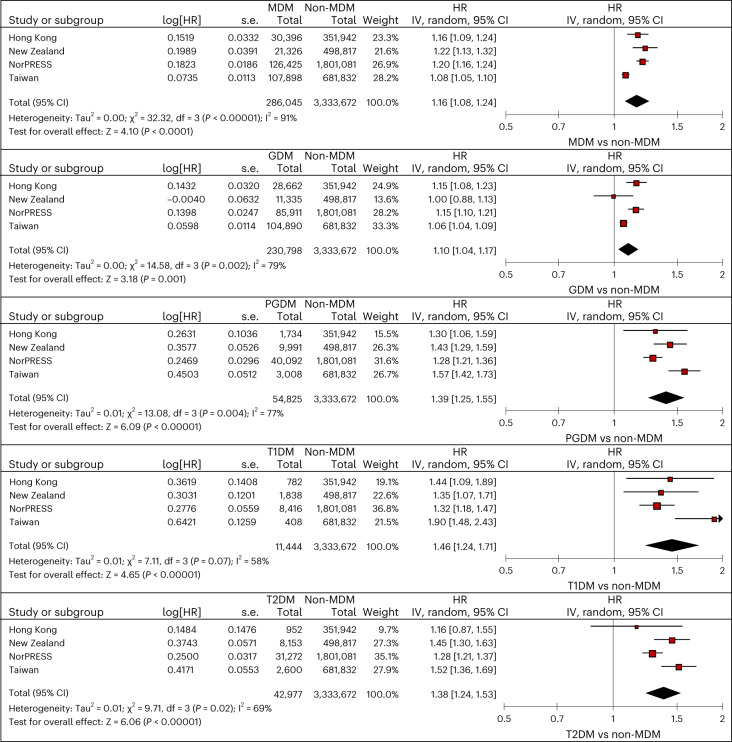
Meta-analyses of maternal diabetes and the risk of ADHD in offspring. Data are presented as HRs and 95% CIs, which were adjusted for demographics, socioeconomic status, birth year, multifetal pregnancies, maternal conditions and use of relevant medications using Cox proportional hazard regression, with a significance level of 5% for a two-sided test. No adjustments were made for multiple comparisons. df, degrees of freedom; IV, inverse variance; s.e., standard error; T1DM, type 1 pregestational diabetes; T2DM, type 2 pregestational diabetes.

**Fig. 4:**
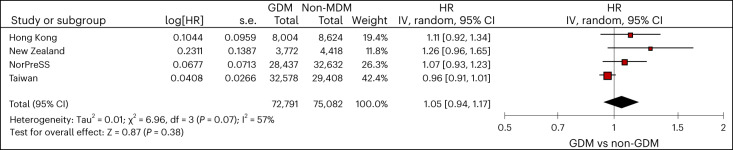
Meta-analyses of discordant GDM exposure in siblings and the risk of ADHD. Data are presented as HRs and 95% CIs, which were adjusted for demographics, socioeconomic status, birth year, multifetal pregnancies, maternal conditions and use of relevant medications using Cox proportional hazard regression, with a significance level of 5% for a two-sided test.

**Extended Data Fig. 3:**
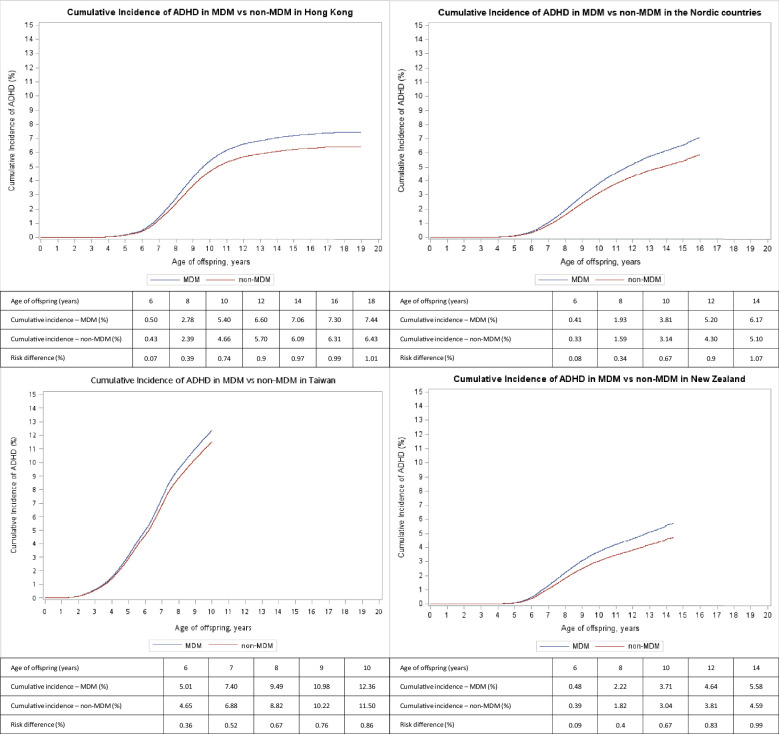
Cumulative incidence of ADHD in different comparison groups from the main analyses. Abbreviations: ADHD, attention-deficit/hyperactivity disorder; MDM, maternal diabetes mellitus.

When comparing children born to mothers with any diabetes during pregnancy (*N*_MDM_ = 286,045) and unexposed children (*N*_non-MDM_ = 3,333,672), we identified a higher risk of ADHD (pooled hazard ratio (HR): 1.16, 95% confidence interval (CI): 1.08-1.24). The cumulative incidence of ADHD for different data sources is shown in Extended Data Fig. 3. Similarly, we identified a higher risk of ADHD across different types of MDM when comparing children whose mothers had GDM (*N*_GDM_ = 230,798), PGDM (*N*_PGDM_ = 54,825), type 1 PGDM (*N*_type 1-PGDM_ = 11,444) and type 2 PGDM (*N*_type 2-PGDM_ = 42,977) to those whose mothers did not have diabetes (GDM pooled HR: 1.10, 95% CI: 1.04 to 1.17; PGDM pooled HR: 1.39, 95% CI: 1.25-1.55, type 1 PGDM pooled HR: 1.46, 95% CI: 1.24-1.71; type 2 PGDM pooled HR: 1.38, 95% CI: 1.24-1.53; Fig. 3 and Table 2). We applied sibling-matched analysis for GDM to control for shared familial confounding including unmeasured lifestyle factors. Siblings who were born to the same mother but with discordant exposure to GDM during the respective pregnancy episodes did not differ in the risks of ADHD (pooled HR: 1.05, 95% CI: 0.94-1.17, *N*_GDM_ = 72,791, N_non-MDM_ = 75,082; Fig. 4 and Table 2). Risks of ADHD for children were similar across those whose mothers had GDM diagnosed at different trimesters in Hong Kong; in Taiwan and New Zealand, the risk of ADHD was highest for children born to mothers with GDM diagnosed in the first trimester (Supplementary Table 4).

**Table 2:** Risks of ADHD in offspring with different maternal diabetes by data sources

	Exposed no. of events/Follow-up time (incidence rate (per 1,000 person years))	Unexposed no. of events/Follow-up time (incidence rate (per 1,000 person years))	PS-weighted HR (95% CI)
**MDM vs non-MDM**			
Hong Kong	1,292/293,721 (4.40)	14,869/4,046,161 (3.67)	**1.16 (1.10-1.24)**
Nordic countries	3,927/1,253,276 (3.13)	45,600/18,450,640 (2.47)	**1.20 (1.16-1.24)**
Taiwan	9,708/828,911 (11.71)	57,841/5,283,760 (10.95)	**1.08 (1.05-1.10)**
New Zealand	659/200,604 (3.29)	13,194/5,142,864 (2.57)	**1.22 (1.13-1.32)**
**GDM vs non-MDM**			
Hong Kong	1,191/274,622 (4.34)	14,869/4,046,161 (3.67)	**1.15 (1.08-1.23)**
Nordic countries	2,528/850,395 (2.97)	45,600/18,450,640 (2.47)	**1.15 (1.10-1.21)**
Taiwan	9,291/806,581 (11.52)	57,841/5,283,760 (10.95)	**1.06 (1.04-1.09)**
New Zealand	265/106,441 (2.49)	13,194/5,142,864 (2.57)	1.00 (0.88-1.13)
**PGDM vs non-MDM**			
Hong Kong	101/19,099 (5.29)	14,869/4,046,161 (3.67)	**1.30 (1.06-1.59)**
Nordic countries	1,380/398,208 (3.47)	45,600/18,450,640 (2.47)	**1.28 (1.21-1.36)**
Taiwan	417/22,330 (18.67)	57,841/5,283,760 (10.95)	**1.57 (1.42-1.73)**
New Zealand	394/94,164 (4.18)	13,194/5,142,864 (2.57)	**1.43 (1.29-1.59)**
**Type 1 PGDM vs non-MDM**			
Hong Kong	53/8,658 (6.12)	14,869/4,046,161 (3.67)	**1.44 (1.09-1.89)**
Nordic countries	310/83,745 (3.70)	45,600/18,450,640 (2.47)	**1.32 (1.18-1.47)**
Taiwan	68/2,998 (22.68)	57,841/5,283,760 (10.95)	**1.90 (1.48-2.43)**
New Zealand	73/18,216 (4.01)	13,194/5,142,864 (2.57)	**1.35 (1.07-1.71)**
**Type 2 PGDM vs non-MDM**			
Hong Kong	48/10,441 (4.60)	14,869/4,046,161 (3.67)	1.16 (0.87-1.55)
Nordic countries	1,052/310,395 (3.39)	45,600/18,450,640 (2.47)	**1.28 (1.21-1.37)**
Taiwan	349/19,332 (18.05)	57,841/5,283,760 (10.95)	**1.52 (1.36-1.69)**
New Zealand	321/75,948 (4.23)	13,194/5,142,864 (2.57)	**1.45 (1.30-1.63)**
**GDM vs PGDM**			
Hong Kong	1,191/274,622 (4.34)	101/19,099 (5.29)	0.84 (0.63-1.13)
Nordic countries	2,528/850,395 (2.97)	1,380/398,208 (3.47)	0.94 (0.86-1.02)
Taiwan	9,291/806,581 (11.52)	417/22,330 (18.67)	**0.65 (0.56-0.76)**
New Zealand	265/106,441 (2.49)	394/94,164 (4.18)	**0.66 (0.56-0.78)**
**Type 2 PGDM vs type 1 PGDM**			
Hong Kong	48/10,441 (4.60)	53/8,658 (6.12)	0.85 (0.53-1.36)
Nordic countries	1,052/310,395 (3.39)	310/83,745 (3.70)	1.12 (0.91-1.38)
Taiwan	349/19,332 (18.05)	68/2,998 (22.68)	0.75 (0.49-1.16)
New Zealand	321/75,948 (4.23)	73/18,216 (4.01)	1.03 (0.74-1.44)
**Medicated GDM vs unmedicated GDM**			
Hong Kong	140/30,467 (4.60)	1,051/244,155 (4.30)	1.21 (0.95-1.55)
Nordic countries	289/87,261 (3.31)	2,239/763,133 (2.93)	1.12 (0.98-1.27)
Taiwan	378/22337 (16.92)	8,913/784,244 (11.37)	**1.41 (1.27-1.57)**
New Zealand	238/99,610 (2.39)	27/6,831 (3.95)	0.62 (0.38-1.01)
**Sibling-matched analyses: GDM vs non-MDM**			
Hong Kong	311/75,607 (4.11)	443/103,958 (4.26)	1.11 (0.92-1.34)
Nordic countries	691/269,892 (2.56)	1,046/36,1821 (2.89)	1.07 (0.93-1.23)
Taiwan	2,799/244,218 (11.46)	2,635/222,656 (11.83)	0.96 (0.91-1.01)
New Zealand	96/33,444 (2.87)	133/48,878 (2.72)	1.26 (0.96-1.65)

HRs in bold indicate statistically significant results.

### Comparisons in different MDM exposures

**Extended Data Fig. 4:**
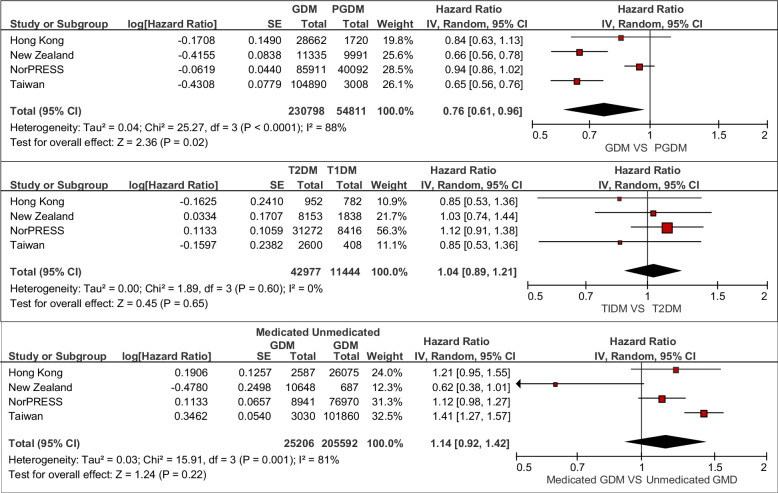
Results of comparisons between different MDM types. Notes: Data are presented as hazard ratios and 95% CIs, which were adjusted for demographics, socioeconomic status, birth year, multifoetal pregnancies, maternal conditions and use of relevant medications using Cox proportional hazard regression, with a significance level of 5% for a two-sided test. No adjustments were made for multiple comparisons. Abbreviations: CI, confidence interval; df, degrees of freedom; GDM, gestational diabetes mellitus; IV, inverse variance; MDM, maternal diabetes mellitus; PGDM, pregestational diabetes mellitus; T1DM, type 1 pregestational diabetes mellitus; T2DM, type 2 pregestational diabetes mellitus; SE, standard error.

Children born to mothers with GDM may have a lower risk of ADHD when compared with children born to mothers with PGDM (pooled HR: 0.76, 95% CI: 0.61-0.96, *N*_GDM_ = 230,798, *N*_PGDM_ = 54,825). Risks of ADHD did not differ between children born to mothers with type 2 PGDM and type 1 PGDM (pooled HR: 1.04, 95% CI: 0.89-1.21, *N*_type 1-PGDM_ = 11,444, *N*_type 2-PGDM_ = 42,977). Children whose mothers had GDM requiring antidiabetic medication had a similar risk of ADHD when compared with children born to unmedicated mothers with GDM (pooled HR: 1.14, 95% CI: 0.92-1.42, *N*_medicated_ = 25,206, *N*_unmedicated_ = 205,592; Table 2 and Extended Data Fig. 4).

### Sensitivity analyses

Results from the sensitivity analyses were similar to the primary analyses when the analytic cohorts included children with less than 6 years of follow-up and were stratified by sex (Supplementary Table 5). We computed E-values to facilitate the interpretation of results in the presence of unmeasured confounding. The E-values of the pooled results range from 1.43 to 2.28, suggesting that unmeasured confounding with an association magnitude equal or greater to both the exposure and outcome could explain away the observed associations respectively (Supplementary Table 6). In other words, any residual confounding is likely to lead to an even smaller estimate. A post hoc analysis also yielded similar results when the analytic cohorts included only children with 9 or more years of follow-up (Supplementary Table 7). Finally, results from the Poisson and negative binomial regression were similar to the Cox regression in the main analysis (Supplementary Table 8).

## Discussion

In this large multinational cohort study, including over 3.6 million mother–child pairs and leveraging a common data model and analytic approach, we found that MDM overall, GDM and PGDM were associated with a small-to-moderate risk of ADHD in offspring. After controlling for shared familial genetic and social factors in the sibling-matched analyses, risks of ADHD did not differ between siblings with discordant exposure to GDM in pregnancy. Due to the discrepancy around the within- and between-family analyses, we speculate that the relationship between GDM exposure and ADHD may be confounded by familial factors.

Compared with a previous meta-analysis which showed a 40% and twofold increased risk of ADHD in children born to mothers with any diabetes and GDM respectively, our study demonstrates a relatively smaller risk estimate of ADHD in children born to mothers with any diabetes after comprehensively controlling for potential confounders. The E-values for the primary analyses ranged from 1.43 to 2.28 and were considered relatively small^32^. Thus, it is likely that there are unmeasured confounders of this magnitude, such as disease severity or paternal factors, that may explain the identified association in our primary analyses. More importantly, although we observed a higher risk of ADHD in offspring whose mothers had GDM at the population level, the association was null in the sibling-matched analysis. Thus, shared familial or genetic factors are likely to at least partly explain the observed association at least between GDM and ADHD in our primary analyses.

In our study, children born to mothers with medicated GDM had a similar risk of ADHD when compared with those with unmedicated GDM. Existing literature regarding the effects of GDM treatment on the risk of ADHD in offspring is scarce. A study using US private healthcare data found a 38% increased risk in children born to mothers requiring treatment for GDM than those with unmedicated GDM^33^. Combining our results with currently available evidence, it remains unclear whether maternal antidiabetic medication during pregnancy could increase the risk of ADHD in offspring or if the severity of GDM requiring antidiabetic medication underlies the association. This further poses a question on the role of glycemic control during pregnancy in mitigating the risk of ADHD in children whose mothers have diabetes.

Our study has limitations. First, most data originated from reimbursement or other administrative purposes rather than research purposes, which represent a variety of data sources, healthcare settings, coding practices, diagnostics criteria, and treatment approaches. Nevertheless, data sources included in this study are all based on high-quality territory-wide electronic health records with robust mother–child linkage and comprehensive information on maternal and child medical records that have been used extensively for pregnancy-related studies^30,34–36^. Moreover, we applied data source-specific identification criteria for not only the exposure and outcome but also the covariates to maximize the comparability of variables included in the study. Second, there may be inaccuracy in coding and incompleteness of diagnoses, prescriptions and laboratory test records. However, we used electronic records and the included pregnant women are likely to have received more frequent clinical monitoring throughout pregnancy. Furthermore, we used a comprehensive set of data ranging from diagnosis records, prescription records and laboratory test records for pregnant women. We defined the study outcome as a diagnosis for ADHD or with ADHD medication prescriptions to capture all possible cases to increase the power of our study, but we acknowledge that misclassification could exist, and some children with minor symptoms of ADHD might have been included in the non-ADHD group. Similarly, nondifferential misclassification of ADHD patients may direct our results towards the null, leading to a smaller effect estimate than previous studies. First, ADHD may not be diagnosed until later in life, which may lead to inclusion of undiagnosed ADHD patients in our non-cases. We therefore conducted a post hoc sensitivity analysis (Supplementary Table 7) to assess the effect of insufficient follow-up duration in our main analyses. After only including mother-baby pairs with at least 9 years of follow-up duration, we found a similar result to the main analyses, demonstrating the robustness of our study conclusion. Similarly, the rates of detection and diagnosis of ADHD were expected to vary with age. This finding implies that the hazards for exposed and unexposed children would naturally exhibit deviations from proportionality throughout the observation period (Extended Data Fig. 3)^8^. We therefore recommend interpreting the HRs we present as a weighted average of the time-varying hazard ratios within our observation period. Nonetheless, sensitivity analyses using Poisson regression and negative binomial regression, which do not rely on the proportional hazards assumption, yielded consistent estimates that support our interpretation and conclusion (Supplementary Table 8). Second, some ADHD medication used in the Nordic countries may be used for other non-ADHD conditions such as narcolepsy. Therefore, ADHD case identification in Nordic countries required at least one ADHD diagnosis and at least two prescription fills for an ADHD medication and thus we were not likely to capture children using ADHD medication for other non-ADHD conditions. Third, maternal lifestyle factors such as physical activity and diet may not be fully captured in electronic health records such that inadequate adjustment for these factors could lead to residual confounding. We thus applied the current study design and analytic approaches to address this concern: 1) the use of sibling-matched analysis for GDM to control for shared familial confounding including lifestyle factors; 2) the computation of E-value to aid the interpretation of the results in presence of unmeasured confounding. For MDM, E-value computation showed that any residual confounding is likely to lead to an even smaller estimate. Therefore, even if these behavioral factors could explain the association, it is unlikely to affect our conclusion that there is a small-to-moderate association between MDM and ADHD, whereas the association between GDM and ADHD is unlikely to be causal. Finally, although the sibling-matched analyses allowed us to control for unmeasured, shared confounding, the design could amplify confounding from factors unique to each sibling^37^. Therefore, we draw our conclusion based on complementary study designs, including the unrelated cohort analyses, sibling-matched comparisons and computation of E-values. More importantly, our results remain robust in all sensitivity analyses and consistent across all data sources from various populations.

It was hypothesized that hyperglycemia may alter the intrauterine environment with increased inflammation, metabolic stress and lipotoxicity, which may affect the neurodevelopment of offspring^6^. Our study, however, only found a small-to-moderate effect between MDM and ADHD where the effect is likely to be confounded by shared genetic and familial factors, at least in the case of GDM. Future studies should explore the specific roles of genetic factors and glycemic control during different developmental stages of the human embryonic brain.

## Methods

### Study design

This is a population-based cohort study with linked mother–child pairs based on healthcare data in Hong Kong, New Zealand, Taiwan and Nordic countries (Finland, Iceland, Norway and Sweden; data nested within the NorPreSS collaboration^38^). The study results were reported following the STrengthening the Reporting of OBservational studies in Epidemiology statement.

All study sites used pseudonymized patient-level electronic health data derived from the respective territory-wide administrative, clinical, or register databases. The list of data sources is provided in Table 1. We applied a distributed network approach with a common data model to harmonize the data structure and standardize the contents from different data sources. Briefly, the coordinating center at the University of Hong Kong distributed a common analytic package for generating aggregated results based on the common data model^38,39^. Site investigators conducted the analyses locally in Hong Kong, New Zealand, Taiwan and Nordic countries where data from individual Nordic countries were pooled into one cohort and analyzed centrally^38^, before sharing aggregated results with the study coordinator. This approach preserved data confidentiality as the individual-level data remained at each site^40^. Moreover, we were able to maintain the consistency of analyses among sites with common analytics^41^. The codes used to identify relevant diagnoses and medication prescriptions from each site are presented in Supplementary Table 8.

### Covariate assessment

**Extended Data Fig. 5:**
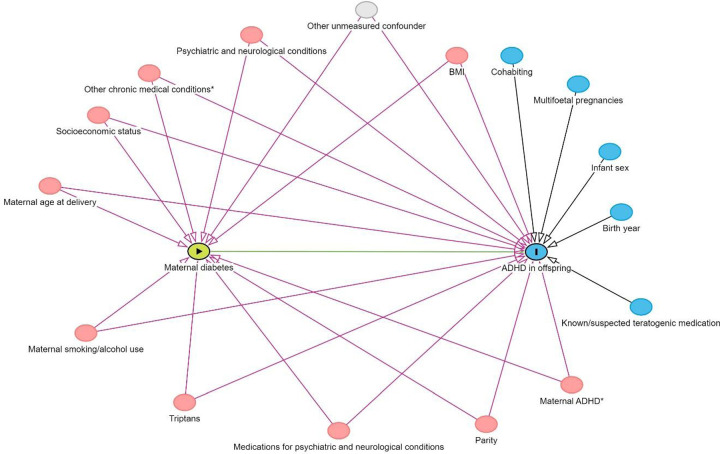
Directed acyclic graph related to the analyses. Notes: * including diagnosis or medication prescription; psychiatric and neurological conditions: including ASD, anxiety disorders, bipolar disorders, depression, disorders of psychological development, epilepsy, illicit drug use, intellectual disability, personality disorders, schizophrenia, sleep disorders; other chronic medical conditions: including cluster headache, crohn’s disease and ulcerative colitis, hypertension, migraine or other headaches, polycystic ovary syndrome, renal disease, rheumatoid arthritis and other inflammatory polyarthropathies, thyroid disorders; Medications for psychiatric and neurological conditions: including antihypertensives, antipsychotics, antidepressants, antiepileptics, antiparkinson drugs, anxiolytics, sedatives, opioids, triptans; socioeconomic status: defined by income level, birth institution or maternal education according to each data source.

Covariates were selected based on known confounders for the study association and risk factors for the study outcomes^30,42–50^, including demographic factors such as maternal age^47,51–53^, infant sex (as directly recorded by clinicians)^54^, socioeconomic status^55,56^, birth year, multifetal pregnancies^57^ and other maternal factors including smoking^44,47,58,59^, alcohol consumption^47,60,61^, psychiatric and neurological conditions^47,48,62–64^, other chronic medical conditions (hypertension^51,65^, renal disease, inflammatory bowel disease^66,67^, autoimmune disease^46,68,69^, thyroid disorders^43,47,70,71^ and polycystic ovary syndrome^45,72,73^), BMI^44,47,51,74^ and use of psychotropic medication^42,47,49^, antihypertensives, ADHD medication and known or suspected teratogenic medication. Various measures were applied as the proxy of socioeconomic status for each data source according to their respective practice (namely, median household income in Hong Kong, education level in Nordic countries, insurance fees in Taiwan and deprivation quintile in New Zealand). Definitions of the covariates by study site are available in Supplementary Table 8. A schematic directed acyclic graph illustrating the causal relationships between the different covariates and the exposure and/or outcome is shown in Extended Data Fig. 5.

### Comparison groups

In our primary analyses, we compared the ADHD status in children born to mothers with any MDM, GDM, any PGDM, type 1 PGDM and type 2 PGDM, with children born to mothers without any diabetes. We also conducted sibling-matched analyses which compared the ADHD status in children born to the same mothers but with discordant GDM exposure. In our secondary analyses, we compared the ADHD status between children born to mothers with different diabetes subtypes, namely GDM and PGDM, type 2 PGDM and type 1 PGDM, and medicated GDM and unmedicated GDM.

### Statistical analysis

We estimated HRs of average treatment effect with 95% CIs to study the associations between MDM status and ADHD using Cox proportional hazard regression models. Propensity score (PS) fine-stratification weighting was used to address the differences in baseline covariates. PS, the probability of receiving treatment conditional on the observed characteristics at baseline, can be applied to account for confounding effects efficiently in observational studies^50,75^. We used PS fine-stratification weighting because of the greater precision, less residual and equivalent bias control compared to traditional PS methods^75,76^. The PS is first used to create 50 fine strata; weights for both exposure and reference patients in all strata are subsequently calculated based on the total number of patients within each stratum, whereas strata with no exposed or reference patients are dropped out before weight calculation^76^. We applied robust standard errors to adjust for data clustering. All the covariates listed in Supplementary Table 8 were included in the PS model. Factors with standardized differences greater than 10% were further adjusted in the Cox models^77^. For missing data, indicator variables for missing maternal characteristics were included in the Nordic (folic acid use, education level, cohabitation, parity, and non-Nordic place of birth) and New Zealand (mother’s socioeconomic status and BMI) models; median imputation was applied for socioeconomic status in Taiwan for missing insurance fees (missing rate = 0.13%).

We conducted sibling-matched analyses to control for shared genetic, familial and environmental confounding factors and used stratified Cox regression with a separate stratum for each family identified by the mother’s unique identification number. Only sibling pairs with discordant exposure and outcome statuses were informative and contributed to the effect estimates.

We pooled the effect estimates from each data source in a meta-analysis using a random-effect model. Meta-analyses were represented in forest plots and the I^2^ statistic was used to quantify heterogeneity between sites. CIs not overlapping 1.0 were considered statistically significant. Statistical Analysis System (SAS) v9.4 (SAS Institute) and R Foundation for Statistical Computing version 3.6.0 were used for data analysis.

### Sensitivity analyses

We conducted sensitivity analyses to test the validity and robustness of the study results. First, we repeated the main analysis in the entire mother-baby cohort, including offspring with less than 6 years of follow-up. Second, we stratified the analyses by offspring’s sex due to a higher prevalence of ADHD in males. Third, to assess the impact of any unmeasured confounders, we computed the E-value, which is defined as the minimum strength of association that an unmeasured confounder would need to have with both exposure and outcome, conditional on the measured covariates, to explain away an observed association^78^. Fourth, a post hoc sensitivity analysis including only mother-baby pairs with at least 9 years of follow-up was conducted to assess the effect of insufficient follow-up duration in our main analyses. Finally, Poisson and negative binomial regression models were also applied in the sensitivity analyses to test the robustness of our results in the presence of model uncertainty.

### Ethics and inclusion statement

The study used healthcare data obtained from Hong Kong, New Zealand, Taiwan and Nordic countries. These data encompassed various sources such as electronic health records, registers, and insurance records. Each participating site followed the relevant local ethics and regulatory frameworks for study approval, namely the Finnish Institute for Health and Welfare (THL/1551/6.02.00/2018, THL/1673/5.05.00/2019) and the Social Insurance Institution of Finland (Kela 148/522/2018 and Kela 117/522/2019) in Finland; the University of Hong Kong/Hospital Authority Hong Kong West Cluster (UW20-051) in Hong Kong; the National Bioethics Committee (VSNb2018060017/03.01) in Iceland; the Norwegian Data Inspectorate (17/02068/Norwegian Data Inspectorate) and the Regional Committee for Medical and Health Research Ethics (2017/2546/REC South-East Norway) in Norway; the New Zealand Health and Disability Ethics Committee (13789) in New Zealand; the Swedish Ethical Review Authority (Dnr 2015/1826-31/2, 2017/2238-32, 2018/1790-32, 2018/2211-32, 2022-04004-02) in Sweden; and the National Cheng Kung University Human Research Ethics Committee (110-453) in Taiwan.

We fully endorse the Nature Portfolio journals’ guidance on authorship and inclusion. All collaborators of this study have fulfilled the criteria for authorship required by Nature Portfolio journals have been included as authors, as their participation was essential for the design and implementation of the study. Roles and responsibilities were agreed among collaborators ahead of the research. This work includes findings that are locally relevant, which have been determined in collaboration with local partners. This research was not severely restricted or prohibited in the setting of the researchers and does not result in stigmatization, incrimination, discrimination or personal risk to participants. Local and regional research relevant to our study was taken into account in citations.

### Reporting summary

Further information on research design is available in the Nature Portfolio Reporting Summary linked to this article.

## Online content

Any methods, additional references, Nature Portfolio reporting summaries, source data, extended data, supplementary information, acknowledgements, peer review information; details of author contributions and competing interests; and statements of data and code availability are available at 10.1038/s41591-024-02917-8.

### Supplementary information

Supplementary Information Supplementary Tables 1–8.

Reporting Summary

## Data Availability

The Hong Kong data used in this study will not be accessible to external parties, as the data custodians have not given permission due to concerns regarding patient privacy protection. Requests can be submitted to hacpaaedr@ha.org.hk (the Central Panel on Administrative Assessment of External Data Requests, the Hospital Authority, Hong Kong). It should be noted that the processing time for such requests may vary as the provided data will be customized for the specific purpose of each project. Individual-level data from the Nordic countries were used under license for the current study and cannot be made publicly available due to data privacy laws. The data are available from the data custodians of the health registers after obtaining the necessary permissions in Finland, Iceland, Norway and Sweden. Due to data privacy laws, individual-level data from the New Zealand cannot be make publicly available. The National Health Research Insurance Database of Taiwan can only be accessed at the Health and Welfare Data Center due to data privacy concerns.

## Code availability

R and SAS codes and sample dataset adopted in this study are available on GitHub repository at https://github.com/legao513/DIAMOND-A (ref. ^79^).
