# Supplementary material for: Maternal diabetes and risk of attention-deficit/hyperactivity disorder in offspring in a multinational cohort of 3.6 million mother–child pairs
Source: Nat Med. 2024 Apr 8;30(5):1416–23. doi: 10.1038/s41591-024-02917-8 (PMC11108779; doi:10.1038/s41591-024-02917-8)
Supplement: Supplementary file 1 — Supplementary Tables 1–8. [file 41591_2024_2917_MOESM1_ESM.pdf]

nature medicine

Article

https://doi.org/10.1038/s41591-024-02917-8

Maternal diabetes and risk of attention-deficit/hyperactivity disorder in offspring in a multinational cohort of 3.6 million mother–child pairs

In the format provided by the authors and unedited

Supplementary Table 1. Mapping codes for exposure, covariates and outcome

Characteristics	Hong Kong	Nordic countries	Taiwan	New Zealand
**Exposure**				
GDM	ICD-9-CM: 648.83. or<br>BNF: 6.1, or<br>Lab test: FPG≥5.1 mmol/L, 2hPG≥8.5mmol/L<br><br>During pregnancy.	NorPreSS algorithm*	⩾1 outpatient records or ⩾1 inpatient records of ICD-9: 6480, 6488, or ICD-10: O244, O249 during pregnancy.<br>OR<br>⩾2 outpatient records or ⩾1 inpatient records of ICD-9: 250, or ICD-10: O24 (excluding O244, O249), E08-E13 during pregnancy.	⩾1 inpatient record of ICD-10-AM: O244, O249, E08-E13<br>OR<br>⩾1 pharmaceutical dispensing record of diabetes medications (insulin and insulin pumps, oral hypoglycaemic agents, acarbose, empagliflozin, sacrosidase and dulaglutide)<br><br>during pregnancy.
PGDM	ICD-9-CM: 250, or<br>BNF: 6.1, or<br>Lab test: FPG≥7.0 mmol/L, 2hPG ≥11.1mmol/L, HbA1c (%) ≥6.5 % (or 48mmol/mol)<br><br>Anytime before pregnancy not including pregnancy period of other pregnancy episodes.<br><br>*If a mother had both GDM and PGDM records for the respective pregnancy episode, the mother will be regarded as having PGDM only and not GDM.	NorPreSS algorithm*	⩾2 outpatient records or ⩾1 inpatient records of ICD-9: 250, or ICD-10: O24 (excluding O244, O249), E08-E13 during (LMP-365 days) to LMP.<br><br>*If a mother met both the criteria of GDM and PGDM, she would be regarded as PGDM but not GDM.	⩾1 inpatient record with a diagnosis ICD-10-AM: O24 (excluding O244, O249), E08-E13<br>or<br>⩾1 pharmaceutical dispensing record of diabetes medications (insulin and insulin pumps, oral hypoglycaemic agents, acarbose, empagliflozin, sacrosidase and dulaglutide) during (LMP-365 days) to LMP.<br><br>*If a mother met both the criteria of GDM and PGDM, she was regarded as PGDM and not GDM.
Type 1-PGDM	ICD-9-CM: 250.01, 250.03, 250.11, 250.13, 250.21, 250.23, 250.31, 250.33, 250.41, 250.43, 250.51, 250.53, 250.61, 250.63, 250.71, 250.73, 250.81, 250.83, 250.91, 250.93, or<br>Drug name: insulin as the first treatment	NorPreSS algorithm*	Among the PGDM patients, if the ICD-9-CM was coded 250.x1, 250.x3 or ICD-10-CM coded O240, E10 then the patient would be regarded as having T1DM.	Among the PGDM patients, if the ICD-10-AM coded O240 or E10 then the patient was regarded as having T1DM.
Type 2-PGDM	Non-type 1 PGDM	NorPreSS algorithm*	Among the PGDM patients, if the ICD-9-CM was coded 250.x0, 250.x2 or ICD-10-CM coded O241, E11 then the patient would be regarded as having T1DM.	Among the PGDM patients, if the ICD-10-AM coded O241 or E11 then the patient was regarded as having T2DM.
**Outcome**				
ADHD	⩾1 patient record of ADHD diagnosis (ICD-9-CM: 314), OR ADHD	⩾2 recorded ICD-10 diagnoses of F90.0 (ADHD, predominantly inattentive type), F90.1 (ADHD,	⩾1 patient record of ADHD diagnosis (outpatient or inpatient; ICD-9: 314, or ICD-10: F90), OR ADHD medications	⩾1 patient record of ADHD diagnosis (inpatient, ICD-10-AM: F90), OR

	medications (BNF: 4.4 atomoxetine and methylphenidate)<br><br>Only ADHD diagnosis or ADHD medication prescription after age of three were used to determine ADHD status.	predominantly hyperactive type), F90.2 (ADHD, combined type),<br><br>OR<br><br>⩾1 recorded ICD-10 diagnosis of F90.0-F90.2 AND ⩾2 prescription fills on different dates for a centrally acting sympathomimetic ADHD drug (ATC: N06BA02 dexamfetamine, N06BA04 methylphenidate, N06BA09 atomoxetine, N06BA12 lisdexamfetamine) or guanfacine (C02AC02).<br><br>Only ADHD diagnosis or ADHD medication prescription after age of three were used to determine ADHD status.	(ATC: N06BA04 methylphenidate and N06BA09 atomoxetine)	ADHD medications (atomoxetine and methylphenidate)<br><br>Only ADHD diagnosis or ADHD medication prescription after age of three were used to determine ADHD status.
**Covariates**				
Maternal age at delivery	As recorded in CDARS	As recorded in MBR	As recorded in NHID	As recorded in the Ministry of Health's Maternity Collection
Infant's sex	As recorded by clinicians in CDARS	As recorded by clinicians in MBR	As recorded by clinicians in NHID	As recorded by clinicians in the Ministry of Health's Maternity Collection
Birth year	As recorded in CDARS	As recorded in MBR	As recorded in NHID	As recorded in the Ministry of Health's Maternity Collection
Parity	As recorded in CDARS	As recorded in MBR	As recorded in NHID	As recorded in the Ministry of Health's Maternity Collection
Multifoetal pregnancies	As recorded in CDARS	As recorded in MBR	As recorded in NHID	As recorded in the Ministry of Health's Maternity Collection
Infant's country of birth	NA	Based on data source: Iceland, Finland, Norway, or Sweden	NA	NA
Available socioeconomic status	Residential district	Highest achieved education in year of delivery<br>Note: Socioeconomic variables were not available from Finland.	Used premium level of mother as proxy	Quintile of New Zealand Deprivation Index[1]
Relationship status/ cohabitation with partner	NA	As recorded in MBR	NA	NA
Smoking	ICD-9-CM: 305.1, V15.82, 649.0, or Smoking records recorded in the patient assessment form from nursing settings	ICD-10: F17, P04.2 (in pregnancy), Z71.6, Z72.0<br>ICPC-1/2: P17<br>ATC: N07BA (drugs used in nicotine dependence, recorded from 365 days before LMP to delivery date)<br>Or as recorded in MBR (Iceland n/a))	ICD-9-CM: 305.1, V15.82, 649.0,<br>or<br>ICD-10: F17, P04.2 (in pregnancy), Z71.6, Z72.0<br>or<br>ATC: N07BA	ICD-10-AM: F17, P04.2 (in pregnancy), Z71.6, Z72.0<br>or<br>ATC: N07BA

Alcohol misuse	ICD-9-CM: 291, 303, 305.0, 357.5, 425.5, 535.3, 571.0, 571.1, 571.2, 571.3, 980, V11.3, or<br>Drinking records recorded in the patient assessment form from nursing settings	ICD-10: E24.4, F10, G31.2, G62.1, G72.1, I42.6, K29.2, K70, K85.2, K86.0, O35.4, P04.3 (in pregnancy), R78.0, T51, Z50.2, Z71.4, Z72.1<br>ICPC-1/2: P15, P16<br>ATC: N07BB (drugs used in alcohol dependence, recorded from 365 days before LMP to delivery date)<br>Reimbursement: Norway: ICD-10: F10.5, K70, ICPC-2: P15.	ICD-9-CM: 291, 303, 305.0, 357.5, 425.5, 535.3, 571.0, 571.1, 571.2, 571.3, 980, V11.3<br>or<br>ICD-10: K70, E24.4, F10, G31.2, G62.1, G72.1, I42.6, K29.2, K85.2, K86.0, O35.4, P04.3 (in pregnancy), R78.0, T51, Z50.2, Z71.4, Z72.1<br>or<br>ATC: N07BB	ICD-10-AM: K70, E24.4, F10, G31.2, G62.1, G72.1, I42.6, K29.2, K85.2, K86.0, O35.4, P04.3 (in pregnancy), R78.0, T51, Z50.2, Z71.4, Z72.1<br>or<br>ATC: N07BB
Antihypertensive	BNF: 2.2, 2.4, 2.5.1, 2.5.2, 2.5.4, 2.5.5, 2.6.2	ATC: C02 (except C02AC02), C03, C07, C08, C09	ATC: C02 (except C02AC02), C03, C07, C08, C09	ATC: C02 (except C02AC02), C03, C07, C08, C09
Antipsychotics	BNF: 4.2	ATC: N05A	ATC: N05A	ATC: N05A
Antidepressants	BNF: 4.3	ATC: N06A	ATC: N06A	ATC: N06A
Antiepileptics	BNF: 4.8	ATC: N03A	ATC: N03A	ATC: N03A
Antiparkinson drugs	BNF: 4.9	ATC: N04	ATC: N04	ATC: N04
Folic acid use	NA	MBR: Recorded in Norway and Sweden,<br>ATC: B03BB01, recorded from 365 days before LMP to delivery date<br><br>No records of folic acid use in PDR or MBR in Finland	ATC: B03BB01	ATC: B03BB01
ADHD medications	BNF: 4.4	ATC: C02AC02, N06BA excl. N06BA05 and N06BA07	ATC: N06BA excl. N06BA05 and N06BA07	ATC: C02AC02, N06BA excl. N06BA05 and N06BA07
Anxiolytics	NA	ATC: N05B	NA	ATC: N05B
Sedatives	BNF: 4.1<br>(including both anxiolytics and sedatives)	ATC: N05C	ATC: N05B, N05C<br>(including both anxiolytics and sedatives)	ATC: N05C
Opioids	BNF: 4.7.2	ATC: N02A	ATC: N02A	ATC: N02A
Teratogenic drugs	BNF: 8.1, 6.2.2, or<br>Drug name: warfarin, orfarin, methotrexat, emthexate, isotretinoin, acnotin, nimegen, oratane, reducar, roaccutane, misopristol, thalidomide, mycophenolic acid, myfortic, leflunomide, arava, aray, lenalidomide, revlimid, pomalidomide, pomalyst, ergot alkaloids	ATC codes of known teratogenic drugs, recorded from 365 days before LMP to delivery date:<br>B01AA03 (warfarin), L01 (antineoplastic agents), L01BA01, L04AX03 (methotrexate), D10AD04, D10BA01, D10AD54 (isotretinoin), D05BB, D11AH04 (systemic retinoids for psoriasis dermatitis), A02BB01, G02AD06, M01AB55, M01AE56 (misopristol), L04AX02 (thalidomide), N03AG01 (valproate)<br>ATC codes of suspected teratogenic drugs, recorded from 365 days before LMP to delivery date:<br>N03A excl. N03AG01 (antiepileptics excl. valproate),	ATC codes of known teratogenic drugs:<br>B01AA03 (warfarin), L01 (antineoplastic agents), L01BA01, L04AX03 (methotrexate), D10AD04, D10BA01, D10AD54 (isotretinoin), D05BB, D11AH04 (systemic retinoids for psoriasis dermatitis), A02BB01, G02AD06, M01AB55, M01AE56 (misopristol), L04AX02 (thalidomide), N03AG01 (valproate)<br>ATC codes of suspected teratogenic drugs:<br>N03 excl. N03AG01 (antiepileptics excl. valproate),<br>N05AN01 (lithium), C09 (drugs acting on the renin-angiotensin system), H03B (antithyroid drugs), L04AA06	ATC codes of known teratogenic drugs:<br>B01AA03 (warfarin), L01 (antineoplastic agents), L01BA01, L04AX03 (methotrexate), D10AD04, D10BA01, D10AD54 (isotretinoin), D05BB, D11AH04 (systemic retinoids for psoriasis dermatitis), A02BB01, G02AD06, M01AB55, M01AE56 (misopristol), L04AX02 (thalidomide), N03AG01 (valproate)<br>ATC codes of suspected teratogenic drugs:<br>N03 excl. N03AG01 (antiepileptics excl. valproate),<br>N05AN01 (lithium), C09 (drugs acting on the renin-angiotensin system), H03B (antithyroid drugs), L04AA06

		N05AN01 (lithium), C09 (drugs acting on the renin-angiotensin system), H03B (antithyroid drugs), L04AA06 (mycophenolic acid), L04AA13 (leflunomide), L04AA31 (teriflunomide), L04AX04 (lenalidomide), L04AX06 (pomalidomide), N02C (ergot alkaloids)	(mycophenolic acid), L04AA13 (leflunomide), L04AX04 (lenalidomide), L04AX06 (pomalidomide), N02C (ergot alkaloids)	(mycophenolic acid), L04AA13 (leflunomide), L04AX04 (lenalidomide), L04AX06 (pomalidomide), N02C (ergot alkaloids)
Triptans	Drug name: triptan	ATC: N02CC	ATC: N02CC	ATC: N02CC
Hypertension	ICD-9-CM: 401-405	ICD-10: I10-I15, O10, O11<br>ICPC-1/2: K86, K87<br>Reimbursement Norway: ICD-10: I10-I15, O10, O11, ICPC-2: K86, K87.<br>Reimbursement Finland: code 205	ICD-9-CM: 401-405<br>Or<br>ICD-10: I10-I15, O10, O11	ICD-10-AM: I10-I15, O10, O11
Renal disease	ICD-9-CM: 580-589, 590-593	ICD-10: N00-N20, N25-N28, T84.2, Y84.1, Z49, Z99.2<br>ICPC-1/2: U70, U88<br>Reimbursement Norway: ICD-10: N00-20, N25, Z99.2, ICPC-2: U70, U88.<br>Reimbursement Finland: codes 137 138 186 306	ICD-9-CM: 580-589, 590-593<br>Or<br>ICD-10: N00-N20, N25-N28, T84.2, Y84.1, Z49, Z99.2	ICD-10-AM: N00-N20, N25-N28, T84.2, Y84.1, Z49, Z99.2
Crohn's disease and ulcerative colitis	ICD-9-CM: 555, 556	ICD-10: K50, K51<br>ICPC-1/2: D94<br>Reimbursement Norway: ICD-10: K50, K51, ICPC-2: D94	ICD-9-CM: 555, 556<br>Or<br>ICD-10: K50 + K51	ICD-10-AM: K50 + K51
Rheumatoid arthritis and other inflammatory polyarthropathies	ICD-9-CM: 714	ICD-10: M05, M06, M08, M45<br>ICPC-1/2: L88<br>Reimbursement Norway: ICD-10: M05, M06, M08, M45, ICPC-2: L88	ICD-9-CM: 714<br>Or<br>ICD-10: M05, M06, M08 M45	ICD-10-AM: M05, M06, M08 M45
Thyroid disorders	ICD-9-CM: 240-246	Hypothyrodism:<br>ICD-10: E01, E03, E89.0<br>ICPC-1/2: T86<br>ATC: H03A, recorded from 365 days before LMP to delivery date<br>Reimbursement: Norway: ICD-10: E03, E89.0, ICPC-2: T86<br>Hyperthyrodism:<br>ICD-10: E05<br>ICPC-1/2: T85<br>ATC: H03B, recorded from 365 days before LMP to delivery date<br>Reimbursement Norway: ICD-10: E05, ICPC-2: T85	ICD-9-CM: 240-246<br>Or<br>ICD-10: E01, E03, E89.0, E05	ICD-10-AM: E01, E03, E89.0, E05
Overweight	ICD-9-CM: 278.02, V85.2, or	$25 \leq BMI < 30$ kg/m^2, calculated from maternal height and weight recorded in MBRs	ICD-9-CM: 278.02, V85.2<br>Or<br>ICD-10: T83	ICD-10-AM: T83

	25≤BMI<30 kg/m² recorded in the family module or the patient assessment form from nursing settings	If missing in MBR:<br>ICD-10: T83		
Obesity	ICD-9-CM: 278.00, 278.01, 278.03, V85.3, V85.4, or<br>BMI≥30 kg/m² recorded in the family module or the patient assessment form from nursing settings	BMI≥30 kg/m², recorded in MBR (height and weight)<br>If missing in MBR:<br>ICD-10: E66<br>ICPC-1/2: T82<br>ATC: A08A (antiobesity preparations, recorded from 365 days before LMP to delivery date)	ICD-9-CM: 278.00, 278.01, 278.03, V85.3, V85.4<br>or<br>ICD-10: E66	ICD-10-AM: E66
Underweight	ICD-9-CM: 783.22, V85.0, or<br>BMI≤18.5 kg/m², recorded in the family module or the patient assessment form from nursing settings	BMI≤18.5 kg/m², recorded in MBR (height and weight)	ICD-9-CM: 783.22, V85.0<br>or<br>ICD10 R63.6	ICD10-AM: R63.6
Polycystic ovary Syndrome	ICD-9-CM: 256.4	ICD-10: E28.2	ICD-9-CM: 256.4<br>Or<br>ICD10 E28.2	ICD10-AM: E28.2
Illicit drug use	ICD-9-CM: 304, 305.2-305.9, 648.3, 760.72, 760.73, 760.75, 292, 965.0, 967.0, 967.6, 967.8, 967.9, 969.6, 969.7, 970.1, 970.81, E850.0, E850.1, E850.2, E852, E854.1, E854.2, E935.0- E935.2, E937, E939.6, E939.7, E940.1, E950.1, E980.2	ICD-10: F11-F16, F18, F19, F55, O35.5, P04.4 (in pregnancy), R78.1, R78.2, R78.3, R78.4, R78.5, T43.6, T43.8, T50.9, Y49, Z50.3, Z71.5, Z72.2, Z86.4<br>ICPC-1/2: P18, P19<br>Reimbursement Norway: ICD-10: F11.5, F12.5, F13.5, F14.5, F15.5, F16.5, F18.5, F19.5, ICPC-2: P18, P19<br>ATC: N07BC (drugs used in opioid dependence, recorded from 365 days before LMP to delivery date)	ICD-9-CM: 304, 305.2-305.9, 648.3, 760.72, 760.73, 760.75, 292, 965.0, 967.0, 967.6, 967.8, 967.9, 969.6, 969.7, 970.1, 970.81, E850.0, E850.1, E850.2, E852, E854.1, E854.2, E935.0- E935.2, E937, E939.6, E939.7, E940.1, E950.1, E980.2<br>Or<br>ICD-10: F11-F16, F18, F19, F55, O35.5, P04.4 (in pregnancy), R78.1, R78.2, R78.3, R78.4, R78.5, T43.6, T43.8, T50.9, Y49, Z50.3, Z71.5, Z72.2, Z86.4	ICD-10-AM: F11-F16, F18, F19, F55, O35.5, P04.4 (in pregnancy), R78.1, R78.2, R78.3, R78.4, R78.5, T43.6, T43.8, T50.9, Y49, Z50.3, Z71.5, Z72.2, Z86.4
Anxiety disorders	ICD-9-CM: 293.84, 300	ICD-10: F40- F42, F44-F48<br>ICPC-1/2: P74, P75, P78, P79<br>Reimbursement Norway: ICD-10: F40.0, F40.1, F41, F41.0, F42, F45, ICPC-2: P74, P79.	ICD-9-CM: 293.84, 300<br>or<br>ICD-10: F40- F42, F44-F48	ICD-10-AM: F40- F42, F44-F48
Depression	ICD-9-CM: 296.2, 296.3, 311, 309.0, 309.1	ICD-10: F32, F33, F34.1, F92.0<br>ICPC-1/2: P76<br>Reimbursement Norway: ICD-10: F32, F33, ICPC-2: P76.	ICD-9-CM: 296.2, 296.3, 311, 309.0, 309.1<br>Or<br>ICD-10: F32, F33, F34.1, F92.0	ICD-10-AM: F32, F33, F34.1, F92.0
Epilepsy	ICD-9-CM: 345	ICD-10: G40, G41<br>ICPC-1/2: N88<br>Reimbursement: Norway: ICD-10: G40, ICPC-2: N88<br>Reimbursement Finland: codes 111, 181, 182, 183, 199.	ICD-9-CM: 345<br>Or<br>ICD-10: G40, G41;	ICD-10-AM: G40, G41
Cluster headache	ICD-9-CM: 339.0	ICD-10: G44.0<br>ICPC-2: N90	ICD-9-CM: 339.0	ICD-10-AM: G44.0

			Or<br>ICD-10: G44.0	
Migraine or other headaches	ICD-9-CM: 346, 784.0	ICD-10: G43, G44.1<br>ICPC-1/2: N89, N90<br>Reimbursement Norway: ICD-10: G43, ICPC-2: N89	ICD-9-CM: 346, 784.0<br>Or<br>ICD-10: G43, G44.1	ICD-10-AM: G43, G44.1
Attention Deficit/Hyperactivity Disorder (ADHD) and autism spectrum disorders (ASD)	ICD-9-CM: 314, 299	ADHD:<br>ICD-10: F90<br>ICPC-2: P81<br>Reimbursement Norway: ICD-10: F90, ICPC-2: P81<br>ASD:<br>ICD-10: F84.0, F84.1, F84.5<br>Reimbursement Norway: ICD-10: F84	ICD-9-CM: 314, 299<br>Or<br>ICD-10: F90, F84.0, F84.1, F84.5	ICD-10-AM: F90, F84.0, F84.1, F84.5
Sleep disorders	ICD-9-CM: 780.5, 347, 327, 307.4	ICD-10: G47<br>ICPC-1/2: P06<br>Reimbursement Norway: ICD-10: G47, ICPC-2: P06	ICD-9-CM: 780.5, 347, 327, 307.4<br>Or<br>ICD-10: G47	ICD-10-AM: G47
Psychotic disorders	ICD-9-CM: 295	ICD-10: F20-F29<br>ICPC-1/2: P72, P98<br>Reimbursement Norway: ICD-10: F20-F25, F28, ICPC-2: P72, P98.	ICD-9-CM: 295<br>Or<br>ICD-10: F20-F29	ICD-10-AM: F20-F29
Bipolar disorders	ICD-9-CM: 296.0, 296.1, 296.4, 296.5, 296.6, 296.7, 296.8	ICD-10: F30, F31<br>ICPC-1/2: P73<br>Reimbursement Norway: ICD-10: F30, F31, ICPC-2: P73, P98.	ICD-9-CM: 296.0, 296.1, 296.4, 296.5, 296.6, 296.7, 296.8<br>Or<br>ICD-10: F30, F31	ICD-10-AM: F30, F31
Intellectual disability	ICD-9-CM: 317-319	ICD-10: F70-F79<br>ICPC-1/2: P85<br>Reimbursement Norway: ICD-10: F70.1, F71.1, F72.1, F73.1, F78.1, F79.1, ICPC-2: P85.	ICD-9-CM: 317-319<br>Or<br>ICD-10: F70.1, F71.1, F72.1, F73.1, F78.1, F79.1	ICD-10-AM: F70.1, F71.1, F72.1, F73.1, F78.1, F79.1
Disorders of psychological development	ICD-9-CM: 315	ICD-10: F80-F83, F88, F89	ICD-9-CM: 315<br>Or<br>ICD-10: F80-F83, F84.2, F84.3, F84.4, F84.8, F84.9, F88, F89	ICD-10-AM: F80-F83, F84.2, F84.3, F84.4, F84.8, F84.9, F88, F89
Personality disorders	ICD-9-CM: 301	ICD-10: F60-F69<br>ICPC-1/2: P80<br>Reimbursement Norway: ICD-10: F61, F62, F63	ICD-9-CM: 301<br>Or<br>ICD-10: F60-F69	ICD-10-AM: F60-F69

Abbreviations: 2hPG, 2-hour post-load plasma glucose; ATC code, Anatomical Therapeutic Chemical code; BNF, British National Formulary; CDARS, Clinical Data Analysis and Reporting System; FPG, fasting plasma glucose; GDM, gestational diabetes mellitus; HbA1c, glycated haemoglobin; ICD-9-CM, International Classification of Diseases, Ninth Revision, Clinical Modification; ICD-10, International Classification of Diseases, Tenth Revision; ICD-10-AM, International Classification of Diseases, Australian Modification Tenth Revision; ICPC-1, International Classification of Primary Care, version 1; ICPC-2, International Classification of Primary Care, version 2; NorPreSS, Nordic Pregnancy Drug Safety Studies; PGDM, pregestational diabetes mellitus.

Unless otherwise stated, sources for the ICD-10 codes were national patient registers (specialist/hospital-based in- and outpatient healthcare), medical birth registers (MBR) and causes of death registers (maternal causes of death not available for Iceland). The source for ICD-10-AM codes was New Zealand's National Minimum Dataset for Hospital Events (NMDS), where diagnoses were from inpatient healthcare. Unless otherwise stated, sources for the ICPC-1 and ICPC-2 codes were primary care, available for Norway since 2006 and Finland since 2012/13. ATC codes were recorded in the prescribed drug registers. Reimbursement codes were recorded in the prescription drug register in Norway (as ICD-10 or ICPC-2 codes) or in the Register of Medical Special Reimbursements in Finland. Unless specified, the lookback period was from 365 days before the last menstrual period (LMP) to delivery date for diagnosis codes and from 365 days before LMP to 1 day before LMP for drug codes used in covariates. For the outcome, only diagnosis codes and drug codes recorded at age 3 or later were considered valid.

1. Atkinson J., Salmond C. and Crampton P. 2014. NZDep2013 Index of Deprivation. Dunedin: University of Otago. https://www.health.govt.nz/publication/nzdep2013-index-deprivation.

*** NorPreSS algorithm for identifying diabetes in pregnancy**

Step 1. Identify any diabetes mellitus among pregnant women
Meets any criterion, LMP-1 year to delivery*
- **MBR (chronic_diabetes, gest_diabetes) – based on check boxes in Norway, Special Refund Entitlement Register in Finland, and ICD-10 codes in all**
- ≥1 diabetes code in **NPR** (ICD-10: E10-E14, O24)
- ≥1 diabetes code in **primary care**** (ICD-10: E10-E14, O24, or ICPC-2: T89-T90, W85)
- ≥1 antidiabetic drug prescription in **PDR***** (ATC A10)

* If the year before LMP includes another pregnancy, ignore diagnoses or prescriptions from a prior pregnancy (applies to steps 1-4)
** Primary care only available for Norway (from 2006) and Finland (from 2012/2013)
*** Exclude if they have zero diabetes diagnoses and metformin as the only antidiabetic, and ART pregnancy (see below) or polycystic ovary syndrome (E282) recorded in NPR/MBR

Step 2. Evaluate for pregestational diabetes mellitus
Meets any criterion:
- **MBR (chronic_diabetes = 1)**
- ≥1 code for any diabetes in **NPR** or **primary care** LMP-1 year to LMP+90 days
- ≥1 antidiabetic drug (A10) prescription in **PDR**, LMP-1 year to LMP+90 days
- ≥1 code for pregestational diabetes mellitus in **NPR** or **primary care** LMP+91 days to delivery

Step 3. Classify pregestational diabetes mellitus

3A. Pregestational diabetes mellitus, type 1 (pG1)
Meets ALL criteria (third criterion ignored if DIABETES_MELLITUS=1 in the second)
- ≥2 insulin prescriptions, LMP-1 year to LMP+90 days
- ≥1 type 1 code in **NPR** (ICD-10: E10, O24.1) LMP-1 year to delivery, or DIABETES_MELLITUS = 1 in **MBRN**
- If codes for both type 1 and 2 and/or other in **NPR** => majority type 1 codes

3B. Pregestational diabetes mellitus, other types (pg0)
Meets ALL criteria
- ≥1 code other than type 1 or 2 in **NPR** (ICD-10: E12-E13, O24.2, O24.3), LMP-1 year to delivery
- If codes for type 1 or 2 are also present => majority codes for other types

3C. Pregestational diabetes mellitus, type 2 (pG2)
Remaining pregestational diabetes mellitus

ART
Meets ANY criterion
- Checkbox in MBRN/MBRS
- ICD-10 code Z311, Z312, Z313 or N98 between LMP-120 and LMP+30 days
- GnRH-analogue + gonadotropin + HCG between LMP-120 and LMP+30 days
  - GnRH: ATC codes H01CA02, H01CC01, H01CC02, L02AE01 or L02AE03
  - Gonadotropin: ATC codes G03GA02, G03GA04, G03GA05, G03GA06, G03GA09 or G03GA30
  - HCG: ATC codes G03GA01 or G03GA08

Step 4. Classify those who did not meet criteria for pregestational diabetes mellitus

4A. Gestational diabetes mellitus (G)
Meets ALL criteria
- ≥1 gestational diabetes code in **NPR/primary care** LMP+91 days to delivery or **MBR** (gest_diabetes = 1)
- No antidiabetic drug prescription before LMP-1 year to LMP+90 days

4B. Diabetes mellitus NOS (N)
All remaining diabetes mellitus

Step 5.
Check for pregestational diabetes mellitus in a prior pregnancy:
- For N or G or pG0 pregnancy, if they had pG2 in any prior pregnancy, reclassify as pG2
- For N, G, pG, if they had a pG1 in any prior or future pregnancy, reclassify as pG1
- For no diabetes mellitus, if they had pG2 in a prior pregnancy, reclassify as pG2

ICD-10 and ICPC-2 codes
Pregestational diabetes mellitus, type 1: ICD-10: O240, E10
Pregestational diabetes mellitus, type 2: ICD-10: O241, E11
Pregestational diabetes mellitus, other: ICD-10: O242, O243, E12, E13
Gestational diabetes mellitus: ICD-10: O244, O249; ICPC-2: W85

Abbreviations
ATC: Anatomical Therapeutic Chemical classification system
GnRH: Gonadotropin-Releasing Hormone
HCG: Human chorionic gonadotropin
ICD-10: International Classification of Diseases, 10th Revision
ICPC-2: International Classification of Primary Care, 2nd Edition
LMP: First day of last menstrual period before pregnancy
MBR: Medical Birth Register
MBRN: Medical Birth Register of Norway
MBRS: Medical Birth Register of Sweden
NPR: National Patient Register (specialist health care, in- and outpatient)
PDR: Prescribed Drug Register

Supplementary Table 2. Characteristics in included mother-child pairs

Characteristics	Hong Kong		Nordic Countries		Taiwan		New Zealand	
	**MDM (N= 30396)**	**Non-MDM (N= 351942)**	**MDM (N=126425)**	**Non-MDM (N=1801081)**	**MDM (N=107898)**	**Non-MDM (N=681832)**	**MDM (N=21326)**	**Non-MDM (N=498817)**
**Children**								
Mean (SD) follow-up time (years)	9.66 (2.91)	11.5 (3.67)	9.91 (2.56)	10.24 (2.56)	7.7 (1.4)	7.7 (1.5)	9.39 (2.36)	10.30 (2.51)
Sex (N, %)								
Girl	14465 (47.6)	169332 (48.1)	61136 (48.4)	879374 (48.8)	51131 (47.4)	329239 (48.3)	10,245 (48.0)	242552 (48.6)
Boy	15931 (52.4)	182610 (51.9)	65289 (51.6)	921707 (51.2)	56767 (52.6)	352593 (51.7)	11,081 (52.0)	256265 (51.4)
Multifoetal pregnancies (N, %)								
Singleton	28635 (94.2)	341489 (97.0)	121672 (96.2%)	174875 (97.1%)	103751 (96.2)	659362 (96.7)	20,540 (96.3)	485364 (97.3)
Multiple	1761 (5.8)	10453 (3.0)	4753 (3.8)	52506 (2.9)	4147 (3.8)	22470 (3.3)	786 (3.7)	13453 (2.7)
**Mothers**								
Mean (SD) maternal age at delivery (years)	34.4 (4.6)	31.2 (5.0)	31.8 (5.4)	30.4 (5.2)	32.6 (4.6)	31.3 (4.7)	32.26 (5.50)	29.64 (6.16)
Parity (N, %)								
0	14037 (46.2)	186425 (53.0)	45509 (36.0)	782293 (43.4)	68173 (63.2)	418585 (61.4)	11,021 (51.7)	262882 (52.7)
1	12189 (40.1)	130316 (37.0)	43734 (34.6)	647372 (35.9)	35402 (32.8)	230099 (33.7)	7,395 (34.7)	170594 (34.2)
2	3158 (10.4)	27691 (7.9)	21494 (17)	254800 (14.1)	4063 (3.8)	30208 (4.4)	2,211 (10.4)	49711 (10.0)
≥3 (3 for Nordics and New Zealand)	1012 (3.3)	7510 (2.1)	8360 (6.6)	71577 (4)	260 (0.2)	2940 (0.4)	540 (2.5)	11684 (2.3)
≥4 (for Nordics and New Zealand)	NA	NA	5052 (4)	36759 (2)	NA	NA	159 (0.7)	3946 (0.8)
Missing	NA	NA	2276 (1.8)	8280 (0.5)	NA	NA	NA	NA
Median household income (HK$) (N, %)					*	*		
<19300	11085 (36.5)	124909 (35.5)	NA	NA	NA	NA	NA	NA
19300-21999	4789 (15.8)	86831 (24.7)	NA	NA	NA	NA	NA	NA
22000-25999	6909 (22.7)	79091 (22.5)	NA	NA	NA	NA	NA	NA
≥26000	7613 (25)	61111 (17.4)	NA	NA	NA	NA	NA	NA
Insurance fee (median, Q1-Q3)	NA	NA	NA	NA	27600 (21900-43900)	25200 (21900-40100)	NA	NA
Maternal highest achieved education							NA	NA
Compulsory	NA	NA	8841 (7.0)	187958 (10.4)	NA	NA	NA	NA
Secondary	NA	NA	17317 (13.7)	549945 (30.5)	NA	NA	NA	NA
Post-secondary	NA	NA	13814 (10.9)	502152 (27.9)	NA	NA	NA	NA
Post-graduate	NA	NA	2460 (1.9)	78618 (4.4)	NA	NA	NA	NA
Missing	NA	NA	83993 (66.4)	482408 (26.8)	NA	NA	NA	NA
Cohabiting with a partner	NA	NA	115497 (91.4)	1636418 (90.9)	NA	NA	NA	NA
Missing	NA	NA	370 (0.3)	4789 (0.3)	NA	NA	-	-
NZ Deprivation Quintile * (N, %)								
1	NA	NA	NA	NA	NA	NA	2,704 (12.7)	70410 (14.1)
2	NA	NA	NA	NA	NA	NA	3,450 (16.2)	82966 (16.6)
3	NA	NA	NA	NA	NA	NA	4,051 (19.0)	92271 (18.5)
4	NA	NA	NA	NA	NA	NA	4,844 (22.7)	104742 (21.0)
5	NA	NA	NA	NA	NA	NA	5,923 (27.8)	135733 (27.2)
Missing	NA	NA	NA	NA	NA	NA	354 (1.7)	12695 (2.6)

Maternal lifestyle factors (N, %)								
Smoking	31 (0.1)	484 (0.1)	17756 (14)	178539 (9.9)	214 (0.2)	1071 (0.2)	2,246 (10.5)	50,919 (10.2)
Alcohol consumption	127 (0.4)	1403 (0.4)	611 (0.5)	5653 (0.3)	143 (0.1)	1078 (0.2)	79 (0.4)	2,135 (0.4)
Maternal medication history (N, %)								
ADHD medications	5 (0)	27 (0)	183 (0.1)	3852 (0.2)	25 (0)	65 (0)	20 (0.1)	358 (0.1)
Antihypertensives	1645 (5.4)	8965 (2.5)	3819 (3)	21667 (1.2)	6420 (6)	32403 (4.8)	1,667 (7.8)	6,930 (1.4)
Antipsychotics	321 (1.1)	2088 (0.6)	1353 (1.1)	10804 (0.6)	7872 (7.3)	49272 (7.2)	806 (3.8)	10,686 (2.1)
Antidepressants	888 (2.9)	5905 (1.7)	7374 (5.8)	86047 (4.8)	2609 (2.4)	15050 (2.2)	2,533 (11.9)	39,739 (8.0)
Antiepileptics	260 (0.9)	1803 (0.5)	1395 (1.1)	14376 (0.8)	1645 (1.5)	9289 (1.4)	230 (1.1)	3,054 (0.6)
Antiparkison drugs	172 (0.6)	1044 (0.3)	88 (0.1)	810 (0)	1597 (1.8)	10298 (1.5)	116 (0.5)	1,592 (0.3)
Anxiolytics	NA	NA	2509 (2)	40374 (2.2)	NA	NA	571 (2.7)	8,285 (1.7)
Folic acid	NA	NA	20344 (16.1)	535854 (29.8)	NA	NA	4,312 (20.2)	52,624 (10.5)
Sedatives	1405 (4.6)	9312 (2.6)	2591 (2)	42376 (2.4)	17778 (16.5)	108320 (15.9)	844 (4.0)	13,560 (2.7)
Opioids	9793 (32.2)	84258 (23.9)	6385 (5.1)	96681 (5.4)	5457 (5.1)	30059 (4.4)	3,839 (18.0)	54,259 (10.9)
Teratogenic drugs	585 (1.9)	4730 (1.3)	6740 (5.3)	67984 (3.8)	4984 (4.6)	26763 (3.9)	1,265 (5.9)	5,914 (1.2)
Triptans	25 (0.1)	192 (0.1)	2326 (1.8)	34350 (1.9)	88 (0.1)	488 (0.1)	376 (1.8)	5,255 (1.1)
Maternal underlying conditions (N, %)								
ADHD or ASD	1 (0)	26 (0)	335 (0.3)	6771 (0.4)	16 (0)	82 (0)	0 (0.0)	9 (0.0)
Anxiety disorders	327 (1.1)	2747 (0.8)	5393 (4.3)	74640 (4.1)	3798 (3.5)	22486 (3.3)	90 (0.4)	847 (0.2)
Bipolar disorders	42 (0.1)	273 (0.1)	764 (0.6)	6053 (0.3)	219 (0.2)	1210 (0.2)	23 (0.1)	241 (0.0)
BMI categories								
Normal	30010 (98.7)	350570 (99.6)	52237 (41.3)	1295334 (71.9)	107,411(99.5)	680,685(99.8)	NA	NA
Obesity	123 (0.4)	141 (0)	38318 (30.3)	150635 (8.4)	487 (0.5) Ɨ	1147 (0.2) Ɨ	499 (2.3)	674 (0.1)
Overweight	199 (0.7)	413 (0.1)	34569 (27.3)	310716 (17.3)	NA	NA	11 (0.1)	226 (0.0)
Underweight	64 (0.2)	818 (0.2)	1301 (1)	44396 (2.5)	8 (0) Ɨ	39 (0) Ɨ	0 (0.0)	0 (0.0)
Cluster headache	NA	NA	29 (0)	289 (0)	NA	NA	NA	NA
Crohn’s disease and ulcerative colitis	7 (0)	65 (0)	881 (0.7)	11483 (0.6)	854 (0.8)	4943 (0.7)	23 (0.1)	371 (0.1)
Depression	455 (1.5)	3059 (0.9)	4233 (3.3)	38397 (2.1)	938 (0.9)	5026 (0.7)	85 (0.4)	890 (0.2)
Disorders of psychological development	2 (0)	16 (0)	57 (0)	433 (0)	<3 (0)	<3 (0)	0 (0.0)	4 (0.0)
Epilepsy	101 (0.3)	967 (0.3)	1012 (0.8)	8863 (0.5)	160 (0.1)	1034 (0.2)	27 (0.1)	517 (0.1)
Hypertension	208 (0.7)	322 (0.1)	5537 (4.4)	16933 (0.9)	1467 (1.4)	3463 (0.5)	464 (2.2)	1,028 (0.2)
Illicit drug use	182 (0.6)	2580 (0.7)	902 (0.7)	11133 (0.6)	95 (0.1)	864 (0.1)	1,815 (8.5)	22,730 (4.6)
Intellectual disability	14 (0)	112 (0)	52 (0)	575 (0)	12 (0)	152 (0)	0 (0.0)	3 (0.0)
Migraine or other headaches	491 (1.6)	3835 (1.1)	2666 (2.1)	31619 (1.8)	11744 (10.9)	71163 (10.4)	105 (0.5)	1,301 (0.3)
Personality disorders	63 (0.2)	548 (0.2)	765 (0.6)	6123 (0.3)	79 (0.1)	441 (0.1)	27 (0.1)	247 (0.1)
Polycystic ovary syndrome	227 (0.7)	440 (0.1)	2086 (1.6)	12707 (0.7)	4394 (4.1)	16889 (2.5)	95 (0.4)	145 (0.0)
Renal disease	393 (1.3)	2946 (0.8)	1410 (1.1)	16367 (0.9)	2567 (2.4)	13746 (2)	340 (1.6)	4855 (1.0)
Rheumatoid arthritis and other inflammatory polyarthropathies	30 (0.1)	175 (0)	783 (0.6)	7572 (0.4)	220 (0.2)	1235 (0.2)	3 (0.0)	42 (0.0)
Schizophrenia	115 (0.4)	677 (0.2)	472 (0.4)	2400 (0.1)	86 (0.1)	495 (0.1)	39 (0.2)	279 (0.1)
Sleep disorders	88 (0.3)	392 (0.1)	333 (0.3)	2376 (0.1)	4191 (3.9)	25105 (3.7)	24 (0.1)	117 (0.0)
Thyroid disorders	494 (1.6)	4159 (1.2)	7979 (6.3)	57189 (3.2)	4115 (3.8)	19972 (2.9)	46 (0.2)	384 (0.1)

Abbreviations: ADHD, attention-deficit/hyperactivity disorder; ASD, autism spectrum disorder; MDM, maternal diabetes mellitus; NA, not applicable; SD, standard deviation. Note: For Taiwan data, numbers less than 3 were too small to report due to data privacy regulations. *For Taiwan data, premium level was applied as the proxy of socioeconomic status. ƗThese covariates were not precisely captured in Taiwan's claim database and was usually resulted in lower-estimation.

Supplementary Table 3. Standardised mean differences (%) before and after PS weighting in different covariates between maternal diabetes exposed and unexposed groups

Variable	Hong Kong		Nordic Countries		Taiwan		New Zealand	
	Before PS weighting	After PS weighting	Before PS weighting	After PS weighting	Before PS weighting	After PS weighting	Before PS weighting	After PS weighting
**Maternal age at delivery**	65.6	-.4	26.3	.9	28.3	-.2	44.8	2.7
**Multifoetal pregnancies**								
Singleton	-13.8	.1	-4.7	-.1	-3.0	.1	-5.8	-0.4
Multiple	13.8	-.1	4.7	.1	3.0	-.1	5.8	0.4
**Infant sex**								
Female	-1.1	.0	-.9	.1	-1.8	.0	-1.2	-0.1
Male	1.1	.0	.9	-.1	1.8	.0	1.2	0.1
**Birth year**								
2001	-11.1	.0	NA	NA	NA	NA	NA	NA
2002	-11.9	-.1	NA	NA	NA	NA	NA	NA
2003	-20.8	-.4	NA	NA	NA	NA	NA	NA
2004	-22.2	-.3	-2.8	.0	NA	NA	NA	NA
2005	-21.8	-.2	-15.6	-.4	NA	NA	NA	NA
2006	-17.2	.2	-3.2	.1	NA	NA	NA	NA
2007	-18.0	.3	-7.2	.2	NA	NA	17.9	2.2
2008	-17.8	.2	-5.6	-.1	NA	NA	19.1	2.5
2009	8.6	-.4	-5.2	-.1	NA	NA	12.7	1.2
2010	11.4	.1	-2.7	-.3	NA	NA	8.4	0.7
2011	13.0	-.1	.8	-.1	-3.0	.1	0.1	0.1
2012	17.1	.1	1.9	-.1	-2.6	.1	2	0.4
2013	17.7	.2	6.3	.3	1.3	.0	10.7	0.9
2014	18.4	.1	23.8	.3	4.2	-.1	16.3	1.1
2015	NA	NA	1.4	.1	NA	NA	17	1.5
**Socioeconomic status**								
**Median household income**								

<19300	2.0	.1	NA	NA	NA	NA	NA	NA
19300-21999	-22.3	.2	NA	NA	NA	NA	NA	NA
22000-25999	.6	-.5	NA	NA	NA	NA	NA	NA
≥26000	18.9	.2	NA	NA	NA	NA	NA	NA
**Insurance fee**	NA	NA	NA	NA	9.2	-.3	NA	NA
**Maternal highest achieved education**							NA	NA
Secondary	NA	NA	-41.4	-.9	NA	NA	NA	NA
Post-secondary	NA	NA	-43.9	-1.0	NA	NA	NA	NA
Post-graduate	NA	NA	-13.9	-.2	NA	NA	NA	NA
Missing	NA	NA	86.6	1.7	NA	NA	NA	NA
**Cohabiting with a partner**	NA	NA	1.8	.2	NA	NA	NA	NA
Missing	NA	NA	.5	.1	NA	NA	NA	NA
**Quintile of Deprivation (NZDep18)**	NA	NA	NA	NA	NA	NA	8.4	1.7
**Parity**								
1	6.3	-.2	-2.8	.1	3.7	.1	1.0	0.8
2	8.8	.0	7.9	.1	-2.0	.0	1.3	0.2
3	5.3	-.1	11.8	.2	-3.4	.0	1.2	1.1
4*	3.9	.2	11.4	.1	-2.9	-.1	0.3	0.3
≥5	3.4	.3	NA	NA	-1.5	-.2	0.5	0.1
Missing	NA	NA	12.7	.2	NA	NA	-	-
**Institution**								
Alice Ho Miu Ling Nethersole Hospital	-1.1	.1	NA	NA	NA	NA	NA	NA
Kwong Wah Hospital	2.6	-.3	NA	NA	NA	NA	NA	NA
Princess Margaret Hospital	10.2	-.5	NA	NA	NA	NA	NA	NA
Prince of Wales Hospital	-25.5	.5	NA	NA	NA	NA	NA	NA
Pamela Youde Nethersole Eastern Hospital	27.3	.9	NA	NA	NA	NA	NA	NA
Queen Elizabeth Hospital	2.4	.4	NA	NA	NA	NA	NA	NA
Queen Mary Hospital	4.0	-1.7	NA	NA	NA	NA	NA	NA
Tuen Mun Hospital	-16.6	.5	NA	NA	NA	NA	NA	NA
Tsan Yuk Hospital	-7.0	-	NA	NA	NA	NA	NA	NA

United Christian Hospital	-3.5	.2	NA	NA	NA	NA	NA	NA
**Country**								
Finland	NA	NA	88.9	1.7	NA	NA	NA	NA
Iceland	NA	NA	-2.4	.0	NA	NA	NA	NA
Norway	NA	NA	-26.6	-.9	NA	NA	NA	NA
Sweden	NA	NA	-67.1	-1.3	NA	NA	NA	NA
Maternal country of birth, Non-Nordic	NA	NA	-5.1	-.6	NA	NA	NA	NA
Maternal country of birth, Missing			18.9	.4			NA	NA
**Maternal lifestyle factors**								
Smoking	-1.0	.0	12.8	.4	1.0	.0	1.1	<0.1
Alcohol consumption	.3	-.1	2.7	.2	-.7	.1	0.9	0.2
**Maternal medication history**								
ADHD medication	.8	.3	-1.6	.1	1.1	.3	0.8	0.1
Antihypertensives	14.7	1.2	12.7	1.3	5.3	.9	31	6.3
Antipsychotics	5.1	.2	5.2	.5	.3	.1	9.7	0.1
Antidepressants	8.3	.3	4.7	.3	1.4	.1	13.1	0.3
Antiepileptics	4.2	.0	3.1	.1	1.4	.1	5.1	<0.1
Antiparkinson drugs	4.1	.4	1.0	.1	.06	.06	3.4	<0.1
Anxiolytics	NA	NA	-1.8	.1	NA	NA	7	<0.1
Folic acid	NA	NA	-32.9	-.9	NA	NA	27	1.6
Folic acid (missing information)	NA	NA	88.9	-1.7	NA	NA	-	-
Sedatives	10.6	.2	-2.1	.1	1.6	.2	6.9	0.1
Opioids	18.5	-.1	-1.4	-.1	3.1	.2	20.4	0.1
Teratogenic drugs	4.6	-.3	7.5	.6	3.4	.6	25.8	6.9
Triptans	1.1	-.1	-.5	-.1	.4	.0	6	0.4
**Maternal underlying conditions**								
ADHD or ASD	-.6	.0	-2.0	.0	.2	.1	0.6	<0.1
Anxiety disorders	3.1	.2	.6	.1	1.2	.1	4.6	0.3
Bipolar disorders	1.8	.1	3.9	.2	.6	.0	2.1	0.5
BMI categories								

Normal	-9.7	-3.0	-64.9	1.4	NA	NA	NA	NA
Obesity	7.7	3.2	57.8	.3	5.1	1.5	20	11.3
Overweight	8.7	1.9	24.4	-1.9	NA	NA	0.3	0.3
Underweight	-.5	.0	-11.0	-.1	.2	.0	NA	NA
Cluster headache #	NA	NA	.5	.0	NA	NA	NA	NA
Crohn's disease and ulcerative colitis	.3	-.2	.7	-.1	.8	.0	1.1	0.2
Depression	5.8	.2	7.5	.3	1.5	.1	4.1	0.3
Disorders of psychological development	.3	.2	1.1	.0	-.3	.0	0.4	<0.1
Epilepsy	1.0	-.2	3.8	-.2	-.1	.0	0.7	0.1
Hypertension	9.5	3.3	21.5	2.6	8.9	2.3	18.2	9.3
Illicit drug use	-1.7	-.2	1.2	.3	-1.2	.0	16.1	1.3
Intellectual disability	.7	.0	.5	.1	-.9	-.1	0.3	<0.1
Migraine or other headaches	4.6	.5	2.6	-.1	1.4	.1	3.8	0.3
Personality disorders	1.2	.3	3.9	.2	.3	.0	2.6	0.4
Polycystic ovary syndrome	9.4	2.1	8.8	-.1	9.0	.0	8.6	4.5
Renal disease	4.4	.2	2.1	.1	2.5	.2	5.5	1.4
Rheumatoid arthritis and other inflammatory polyarthropathies	1.8	-.1	2.8	.0	.5	.0	0.5	0.4
Schizophrenia	3.5	.3	4.8	.5	.3	.1	3.7	0.8
Sleep disorders	4.0	.7	3.0	.5	1.1	.1	3.4	1.4
Thyroid disorders	3.8	.0	14.8	.2	4.9	.1	3.6	0.5

Abbreviation: ADHD, attention-deficit/hyperactivity disorder; ASD, autism spectrum disorder; NA, not applicable; PS, propensity score.

* For Nordic countries, parity was included as five categorical classes: 0, 1, 2, 3, ⩾4; # For Nordic countries, migraine and cluster headache were included as separate variables in the PS model whereas in Hong Kong and Taiwan, migraine and cluster headache were included as one variable - "migraine or other headaches".

Supplementary Table 4. Results of GDM diagnosed at different trimesters and non-MDM

		Exposed	Unexposed	PS-weighted
		No. of events/ Follow-up time (incidence rate [per 1000 person years]))	No. of events/ Follow-up time (incidence rate [per 1000 person years]))	HR (95% CI)
Hong Kong				
	Trimester 1	37/7930 (4.67)	14869/4046161 (3.67)	1.25 (0.90-1.74)
	Trimester 2	219/50129 (4.37)	14869/4046161 (3.67)	**1.19 (1.03-1.37)**
	Trimester 3	935/216562 (4.32)	14869/4046161 (3.67)	**1.14 (1.06-1.22)**
Taiwan				
	Trimester 1	129/7566 (17.05)	57841/5283760 (10.95)	**1.54 (1.29-1.83)**
	Trimester 2	2892/244488 (11.83)	57841/5283760 (10.95)	**1.09 (1.05-1.13)**
	Trimester 3	6270/554527 (11.31)	57841/5283760 (10.95)	**1.04 (1.02-1.07)**
New Zealand				
	Trimester 1	20/6310 (3.17)	13194/5142864 (2.57)	1.19 (0.77-1.84)
	Trimester 2	26/14669 (1.77)	13194/5142864 (2.57)	0.75 (0.51-1.10)
	Trimester 3	219/85462 (2.56)	13194/5142864 (2.57)	1.03 (0.90-1.18)

Notes: The bolded numbers for HRs indicate statistically significant results. Abbreviations: CI, confidence interval; GDM, gestational diabetes mellitus; HR, hazard ratio; MDM, maternal diabetes mellitus; PS, propensity score.

Supplementary Table 5. Sensitivity analyses: without follow-up time limitation and stratified by sexes

	Exposed	Unexposed	PS-weighted
	No. of events/ Follow-up time (incidence rate [per 1000 person years]))	No. of events/ Follow-up time (incidence rate [per 1000 person years]))	HR (95% CI)
MDM vs non-MDM			
Total			
Hong Kong	1322/361332 (3.66)	15012/4595295 (3.27)	**1.16 (1.09-1.24)**
Nordic countries	4516/1519531 (2.97)	51461/22007033 (2.34)	**1.22 (1.17-1.26)**
Taiwan	12280/1228057 (10.00)	73991/8316650 (8.90)	**1.08 (1.06-1.10)**
New Zealand	684/274302 (2.49)	13372/6113200 (2.19)	**1.26 (1.17-1.37)**
Pooled	18802/3383222 (5.56)	153836/41032178 (3.75)	**1.17 (1.08-1.27)**
Boys			
Hong Kong	1012/189085 (5.35)	11576/2369928 (4.88)	**1.16 (1.09-1.25)**
Nordic Countries	3467/783626 (4.42)	38846/11244468 (3.45)	**1.20 (1.15-1.24)**
Taiwan	9375/639552 (14.66)	56044/4263927 (13.14)	**1.08 (1.05-1.10)**
New Zealand	510/141928 (3.59)	10469/3127624 (3.35)	**1.21 (1.11-1.33)**
Pooled	14364/1754191 (8.19)	116935/21005947 (5.57)	**1.15 (1.08-1.24)**
Girls			
Hong Kong	310/172247 (1.80)	3436/2225366 (1.54)	**1.19 (1.05-1.35)**
Nordic Countries	1049/735905 (1.43)	12615/10762565 (1.17)	**1.30 (1.21-1.39)**
Taiwan	2905/588505 (4.94)	17947/4052722 (4.43)	**1.08 (1.04-1.12)**
New Zealand	174/132375 (1.31)	2903/2985576 (0.97)	**1.45 (1.24-1.70)**
Pooled	4438/1629032 (2.72)	36901/20026229 (1.84)	**1.24 (1.08-1.41)**
GDM vs non-MDM			
Total			
Hong Kong	1219/338939 (3.60)	15012/4595295 (3.27)	**1.16 (1.09-1.23)**
Nordic Countries	2906/1029452 (2.82)	51461/22007033 (2.34)	**1.17 (1.13-1.22)**
Taiwan	11715/1189041 (9.85)	73991/8316650 (8.90)	**1.06 (1.04-1.08)**
New Zealand	278/148987 (1.87)	13372/6113200 (2.19)	1.03 (0.91-1.16)
Pooled	16118/2706419 (5.96)	153836/41032178 (3.75)	**1.09 (1.07-1.11)**
Boys			
Hong Kong	933/177557 (5.25)	11576/2369928 (4.88)	**1.16 (1.08-1.24)**
Nordic Countries	2283/532765 (4.29)	38846/11244468 (3.45)	**1.16 (1.11-1.22)**
Taiwan	8968/619816 (14.47)	56044/4263927 (13.14)	**1.06 (1.04-1.09)**
New Zealand	215/78839 (2.73)	10469/3127624 (3.35)	0.99 (0.87-1.14)
Pooled	12399/1408977 (8.80)	116935/21005947 (5.57)	**1.10 (1.04-1.18)**
Girls			
Hong Kong	286/161382 (1.77)	3436/2225366 (1.54)	**1.19 (1.04-1.35)**
Nordic Countries	623/496687 (1.25)	12615/10762565 (1.17)	**1.22 (1.12-1.33)**
Taiwan	2747/569225 (4.83)	17947/4052722 (4.43)	**1.06 (1.02-1.10)**
New Zealand	63/70149 (0.90)	2903/2985576 (0.97)	1.13 (0.87-1.45)
Pooled	3719/1297443 (2.87)	36901/20026229 (1.84)	**1.14 (1.04-1.25)**
PGDM vs non-MDM			
Total			
Hong Kong	103/22393 (4.60)	15012/4595295 (3.27)	**1.25 (1.02-1.53)**
Nordic Countries	1589/484781 (3.28)	51461/22007033 (2.34)	**1.30 (1.23-1.36)**
Taiwan	565/39016 (14.48)	73991/8316650 (8.90)	**1.54 (1.41-1.68)**
New Zealand	406/125315 (3.24)	13372/6113200 (2.19)	**1.48 (1.34-1.64)**
Pooled	2663/671505 (3.97)	153836/41032178 (3.75)	**1.40 (1.26-1.55)**
Boys			
Hong Kong	79/11528 (6.85)	11576/2369928 (4.88)	**1.26 (1.00-1.59)**
Nordic Countries	1168/248061 (4.71)	38846/11244468 (3.45)	**1.26 (1.19-1.34)**
Taiwan	407/19736 (20.62)	56044/4263927 (13.14)	**1.51 (1.37-1.67)**
New Zealand	295/63089 (4.68)	10469/3127624 (3.35)	**1.42 (1.26-1.60)**
Pooled	1949/342414 (5.69)	116935/21005947 (5.57)	**1.37 (1.23-1.52)**
Girls			
Hong Kong	24/10865 (2.21)	3436/2225366 (1.54)	1.18 (0.77-1.81)
Nordic Countries	421/236720 (1.78)	12615/10762565 (1.17)	**1.43 (1.29-1.58)**
Taiwan	158/19280 (8.20)	17947/4052722 (4.43)	**1.70 (1.45-1.99)**
New Zealand	111/62226 (1.78)	2903/2985576 (0.97)	**1.70 (1.39-2.07)**
Pooled	714/329091 (2.17)	36901/20026229 (1.84)	**1.54 (1.36-1.74)**
Type 1-PGDM vs non-MDM			

Total			
Hong Kong	53/10025 (5.29)	15012/4595295 (3.27)	**1.40 (1.06-1.85)**
Nordic Countries	364/101706 (3.58)	51461/22007033 (2.34)	**1.35 (1.22-1.50)**
Taiwan	87/5564 (15.64)	73991/8316650 (8.90)	**1.76 (1.42-2.19)**
New Zealand	76/24249 (3.13)	13372/6113200 (2.19)	**1.44 (1.14-1.80)**
Pooled	580/141544 (4.10)	153836/41032178 (3.75)	**1.45 (1.29-1.63)**
Boys			
Hong Kong	39/5275 (7.39)	11576/2369928 (4.88)	1.30 (0.94-1.79)
Nordic Countries	276/52375 (5.27)	38846/11244468 (3.45)	**1.36 (1.21-1.53)**
Taiwan	64/2746 (23.31)	56044/4263927 (13.14)	**1.85 (1.44-2.37)**
New Zealand	56/12259 (4.57)	10469/3127624 (3.35)	**1.41 (1.08-1.83)**
Pooled	435/72655 (5.99)	116935/21005947 (5.57)	**1.45 (1.26-1.68)**
Girls			
Hong Kong	14/4751 (2.95)	3436/2225366 (1.54)	1.69 (0.99-2.91)
Nordic Countries	88/49331 (1.78)	12615/10762565 (1.17)	**1.34 (1.08-1.65)**
Taiwan	23/2817 (8.16)	17947/4052722 (4.43)	**1.73 (1.15-2.59)**
New Zealand	20/11990 (1.67)	2903/2985576 (0.97)	**1.57 (1.01-2.45)**
Pooled	145/68889 (2.10)	36901/20026229 (1.84)	**1.46 (1.24-1.72)**
Type 2-PGDM vs non-MDM			
Total			
Hong Kong	50/12367 (4.04)	15012/4595295 (3.27)	1.10 (0.82-1.46)
Nordic Countries	1203/378260 (3.18)	51461/22007033 (2.34)	**1.29 (1.21-1.37)**
Taiwan	478/33453 (14.29)	73991/8316650 (8.90)	**1.51 (1.37-1.65)**
New Zealand	330/101066 (3.27)	13372/6113200 (2.19)	**1.50 (1.34-1.68)**
Pooled	2061/525146 (3.92)	153836/41032178 (3.75)	**1.38 (1.24-1.54)**
Boys			
Hong Kong	40/6254 (6.40)	11576/2369928 (4.88)	1.23 (0.89-1.69)
Nordic Countries	874/193181 (4.52)	38846/11244468 (3.45)	**1.23 (1.15-1.32)**
Taiwan	343/16990 (20.19)	56044/4263927 (13.14)	**1.45 (1.30-1.62)**
New Zealand	239/50830 (4.70)	10469/3127624 (3.35)	**1.42 (1.25-1.62)**
Pooled	1496/267255 (5.60)	116935/21005947 (5.57)	**1.34 (1.21-1.49)**
Girls			
Hong Kong	10/6114 (1.64)	3436/2225366 (1.54)	0.79 (0.41-1.55)
Nordic Countries	329/185080 (1.78)	12615/10762565 (1.17)	**1.48 (1.32-1.65)**
Taiwan	135/16463 (8.20)	17947/4052722 (4.43)	**1.68 (1.41-2.00)**
New Zealand	91/50236 (1.81)	2903/2985576 (0.97)	**1.73 (1.39-2.15)**
Pooled	565/257893 (2.19)	36901/20026229 (1.84)	**1.55 (1.34-1.80)**
GDM vs PGDM			
Total			
Hong Kong	1219/338939 (3.60)	103/22393 (4.60)	0.73 (0.52-1.03)
Nordic Countries	2906/1029452 (2.82)	1589/484781 (3.28)	**0.92 (0.85-0.99)**
Taiwan	11715/1189041 (9.85)	565/39016 (14.48)	**0.67 (0.59-0.76)**
New Zealand	278/148987 (1.87)	406/125315 (3.24)	**0.65 (0.55-0.77)**
Pooled	16118/2706419 (5.96)	2663/671505 (3.97)	**0.74 (0.60-0.92)**
Boys			
Hong Kong	933/177557 (5.25)	79/11528 (6.85)	0.96 (0.66-1.40)
Nordic Countries	2283/532765 (4.29)	1168/248061 (4.71)	0.95 (0.87-1.04)
Taiwan	8968/619816 (14.47)	407/19736 (20.62)	**0.68 (0.59-0.78)**
New Zealand	215/78839 (2.73)	295/63089 (4.68)	**0.66 (0.55-0.80)**
Pooled	12399/1408977 (8.80)	1949/342414 (5.69)	**0.79 (0.63-0.99)**
Girls			
Hong Kong	286/161382 (1.77)	24/10865 (2.21)	**0.53 (0.28-0.99)**
Nordic Countries	623/496687 (1.25)	421/236720 (1.78)	**0.81 (0.69-0.95)**
Taiwan	2747/569225 (4.83)	158/19280 (8.20)	**0.64 (0.51-0.80)**
New Zealand	63/70149 (0.90)	111/62226 (1.78)	**0.64 (0.45-0.89)**
Pooled	3719/1297443 (2.87)	714/329091 (2.17)	**0.70 (0.59-0.83)**
Type 2-PGDM vs Type 1-PGDM			
Total			
Hong Kong	50/12367 (4.04)	53/10025 (5.29)	1.03 (0.67-1.61)
Nordic Countries	1203/378260 (3.18)	364/101706 (3.58)	1.06 (0.87-1.30)
Taiwan	478/33453 (14.29)	87/5564 (15.64)	0.91 (0.65-1.29)
New Zealand	330/101066 (3.27)	76/24249 (3.13)	1.02 (0.75-1.38)
Pooled	2061/525146 (3.92)	580/141544 (4.10)	1.02 (0.89-1.18)
Boys			
Hong Kong	40/6254 (6.40)	39/5275 (7.39)	1.20 (0.72-2.00)
Nordic Countries	874/193181 (4.52)	276/52375 (5.27)	1.07 (0.85-1.34)

Taiwan	343/16990 (20.19)	64/2746 (23.31)	0.71 (0.44-1.14)
New Zealand	239/50830 (4.70)	56/12259 (4.57)	0.99 (0.69-1.42)
Pooled	1496/267255 (5.60)	435/72655 (5.99)	1.01 (0.85-1.20)
Girls			
Hong Kong	10/6114 (1.64)	14/4751 (2.95)	0.38 (0.16-0.92)
Nordic Countries	329/185080 (1.78)	88/49331 (1.78)	1.06 (0.72-1.56)
Taiwan	135/16463 (8.20)	23/2817 (8.16)	0.91 (0.49-1.67)
New Zealand	91/50236 (1.81)	20/11990 (1.67)	1.23 (0.70-2.17)
Pooled	565/257893 (2.19)	145/68889 (2.10)	0.92 (0.63-1.34)

Notes: The bolded numbers for HRs indicate statistically significant results. Abbreviations: CI, confidence interval; HR, hazard ratio; GDM, gestational diabetes mellitus; MDM, maternal diabetes mellitus; PGDM, pregestational diabetes mellitus; PS, propensity score.

Supplementary Table 6. Sensitivity analyses: E-values of main analyses

	Pooled PS-weighted	E-value*
	HR (95% CI)	
MDM vs non-MDM	**1.16 (1.08-1.24)**	**1.59**
GDM vs non-MDM	**1.10 (1.04-1.17)**	**1.43**
PGDM vs non-MDM	**1.39 (1.25-1.55)**	**2.13**
T1DM vs non-MDM	**1.46 (1.24-1.71)**	**2.28**
T2DM vs non-MDM	**1.38 (1.24-1.53)**	**2.10**
GDM vs PGDM	**0.76 (0.61-0.96)**	**1.96**
T2DM vs T1DM	1.04 (0.89-1.21)	/
Medicated GDM vs unmedicated GDM	1.14 (0.92-1.42)	/

Notes: The bolded numbers for HRs and E-value indicate statistically significant results. Abbreviations: CI, confidence interval; HR, hazard ratio; GDM, gestational diabetes mellitus; MDM, maternal diabetes mellitus; PGDM, pregestational diabetes mellitus; PS, propensity score; T1DM, type 1 diabetes mellitus; T2DM, type 2 diabetes mellitus.

* - E-value is defined as the minimum strength of association that an unmeasured confounder would need to have with both treatment and outcome, conditional on the measured covariates, to explain away an observed association.[1]

Reference

1. VanderWeele TJ, Ding P. Sensitivity Analysis in Observational Research: Introducing the E-Value. Ann Intern Med 2017;167:268-74.

Supplementary Table 7. Post-hoc analyses: with at least 9 years of follow-up time (if data is up to 2020, then include children born <= 2011)

	**Exposed**	**Unexposed**	**PS-weighted**
	No. of events/ Follow-up time (incidence rate [per 1000 person years]))	No. of events/ Follow-up time (incidence rate [per 1000 person years]))	HR (95% CI)
MDM vs non-MDM			
Hong Kong	942/187590 (5.02)	12962/3275117 (3.96)	**1.15 (1.07-1.23)**
Nordic countries	1446/854632 (1.69)	20402/13511585 (1.51)	**1.14 (1.07-1.21)**
Taiwan	2620/220182 (11.90)	16162/1474321 (10.96)	**1.10 (1.05-1.14)**
New Zealand	326/107000 (3.05)	7665/3611452 (2.12)	**1.32 (1.18-1.48)**
Pooled	5334/1369404 (3.90)	57191/21872475 (2.61)	**1.15 (1.09-1.23)**
GDM vs non-MDM			
Hong Kong	861/172810 (4.98)	12962/3275117 (3.96)	**1.14 (1.06-1.22)**
Nordic countries	887/576638 (1.54)	20402/13511585 (1.51)	1.06 (0.99-1.14)
Taiwan	2501/214331 (11.67)	16162/1474321 (10.96)	**1.08 (1.03-1.12)**
New Zealand	128/55364 (2.31)	7665/3611452 (2.12)	1.09 (0.91-1.30)
Pooled	4377/1019143 (4.29)	57191/21872475 (2.61)	**1.09 (1.05-1.12)**
PGDM vs non-MDM			
Hong Kong	81/14780 (5.48)	12962/3275117 (3.96)	1.25 (0.99-1.57)
Nordic countries	550/274143 (2.01)	20402/13511585 (1.51)	**1.28 (1.17-1.40)**
Taiwan	119/5851 (20.34)	16162/1474321 (10.96)	**1.81 (1.49-2.19)**
New Zealand	198/51636 (3.83)	7665/3611452 (2.12)	**1.51 (1.30-1.75)**
Pooled	948/346410 (2.74)	57191/21872475 (2.61)	**1.44 (1.23-1.68)**
Type 1-PGDM vs non-MDM			
Hong Kong	44/6645 (6.62)	12962/3275117 (3.96)	**1.47 (1.08-2.00)**
Nordic countries	120/58384 (2.06)	20402/13511585 (1.51)	**1.21 (1.01-1.45)**
Taiwan	22/806 (27.30)	16162/1474321 (10.96)	**2.50 (1.54-4.07)**
New Zealand	40/11672 (3.43)	7665/3611452 (2.12)	**1.41 (1.02-1.93)**
Pooled	226/77507 (2.92)	57191/21872475 (2.61)	**1.48 (1.16-1.89)**
Type 2-PGDM vs non-MDM			
Hong Kong	37/8135 (4.55)	12962/3275117 (3.96)	1.06 (0.76-1.48)
Nordic countries	421/213042 (1.98)	20402/13511585 (1.51)	**1.29 (1.17-1.43)**
Taiwan	97/5045 (19.23)	16162/1474321 (10.96)	**1.69 (1.38-2.09)**
New Zealand	158/39964 (3.95)	7665/3611452 (2.12)	**1.52 (1.29-1.79)**
Pooled	713/266186 (2.68)	57191/21872475 (2.61)	**1.40 (1.20-1.64)**
GDM vs PGDM			
Hong Kong	861/172810 (4.98)	81/14780 (5.48)	0.69 (0.46-1.02)
Nordic countries	887/576638 (1.54)	550/274143 (2.01)	**0.83 (0.72-0.95)**
Taiwan	2501/214331 (11.67)	119/5851 (20.34)	**0.60 (0.44-0.81)**
New Zealand	128/55364 (2.31)	198/51636 (3.83)	**0.67 (0.52-0.85)**
Pooled	4377/1019143 (4.29)	948/346410 (2.74)	**0.72 (0.61-0.84)**
Type 2-PGDM vs Type 1-PGDM			
Hong Kong	37/8135 (4.55)	44/6645 (6.62)	0.80 (0.47-1.35)
Nordic countries	421/213042 (1.98)	120/58384 (2.06)	1.21 (0.85-1.71)
Taiwan	97/5045 (19.23)	22/806 (27.30)	0.65 (0.33-1.29)
New Zealand	158/39964 (3.95)	40/11672 (3.43)	1.20 (0.80-1.80)
Pooled	713/266186 (2.68)	226/77507 (2.92)	1.02 (0.78-1.33)

Notes: The bolded numbers for HRs indicate statistically significant results. Abbreviations: CI, confidence interval; HR, hazard ratio; GDM, gestational diabetes mellitus; MDM, maternal diabetes mellitus; PGDM, pregestational diabetes mellitus; PS, propensity score; T1DM, type 1 diabetes mellitus; T2DM, type 2 diabetes mellitus.

Supplementary Table 8. Sensitivity analyses: different regression models

	**Poisson regression**	**Negative binomial regression**
	PS-weighted IRR （95% CI）	PS-weighted IRR （95% CI）
MDM vs non-MDM		
Hong Kong	**1.16 (1.09-1.23)**	**1.16 (1.09-1.23)**
Nordic countries	**1.21 (1.17-1.25)**	**1.21 (1.17-1.25)**
Taiwan	**1.07 (1.05-1.09)**	**1.07 (1.05-1.09)**
New Zealand	**1.23 (1.13-1.33)**	**1.23 (1.13-1.33)**
Pooled	**1.16 (1.07-1.26)**	**1.16 (1.07-1.26)**
GDM vs non-MDM		
Hong Kong	**1.15 (1.08-1.22)**	**1.15 (1.08-1.22)**
Nordic countries	**1.16 (1.12-1.21)**	**1.16 (1.12-1.21)**
Taiwan	**1.06 (1.03-1.08)**	**1.06 (1.03-1.08)**
New Zealand	1.01 (0.89-1.14)	1.01 (0.89-1.14)
Pooled	**1.10 (1.03, 1.18)**	**1.10 (1.03, 1.18)**
PGDM vs non-MDM		
Hong Kong	**1.30 (1.06-1.58)**	**1.30 (1.06-1.58)**
Nordic countries	**1.29 (1.22-1.36)**	**1.29 (1.22-1.36)**
Taiwan	**1.50 (1.36-1.65)**	**1.50 (1.36-1.65)**
New Zealand	**1.42 (1.29-1.57)**	**1.42 (1.29-1.57)**
Pooled	**1.38 (1.27, 1.50)**	**1.38 (1.27, 1.50)**

Notes: The bolded numbers for HRs indicate statistically significant results. Abbreviations: CI, confidence interval; GDM, gestational diabetes mellitus; IRR, incidence rate ratio; MDM, maternal diabetes mellitus; PGDM, pregestational diabetes mellitus; PS, propensity score.
