# Supplementary material for: Maternal diabetes and risk of attention-deficit/hyperactivity disorder in offspring in a multinational cohort of 3.6 million mother–child pairs
Source: Nat Med. 2024 Apr 8;30(5):1416–23. doi: 10.1038/s41591-024-02917-8 (PMC11108779; doi:10.1038/s41591-024-02917-8)
Supplement: Supplementary file 2 — Reporting Summary [file 41591_2024_2917_MOESM2_ESM.pdf]

nature portfolio

Corresponding author(s): Carolyn E Cesta, Edward Chia-Cheng Lai, Helga Zoega, Kenneth KC Man, Ian CK Wong
NMED-A128377B-Z

Last updated by author(s): Mar 1, 2024

Reporting Summary

Nature Portfolio wishes to improve the reproducibility of the work that we publish. This form provides structure for consistency and transparency in reporting. For further information on Nature Portfolio policies, see our Editorial Policies and the Editorial Policy Checklist.

Statistics

For all statistical analyses, confirm that the following items are present in the figure legend, table legend, main text, or Methods section.

n/a	Confirmed	
☐	☒	The exact sample size (*n*) for each experimental group/condition, given as a discrete number and unit of measurement
☒	☐	A statement on whether measurements were taken from distinct samples or whether the same sample was measured repeatedly
☐	☒	The statistical test(s) used AND whether they are one- or two-sided<br>*Only common tests should be described solely by name; describe more complex techniques in the Methods section.*
☐	☒	A description of all covariates tested
☐	☒	A description of any assumptions or corrections, such as tests of normality and adjustment for multiple comparisons
☐	☒	A full description of the statistical parameters including central tendency (e.g. means) or other basic estimates (e.g. regression coefficient) AND variation (e.g. standard deviation) or associated estimates of uncertainty (e.g. confidence intervals)
☐	☒	For null hypothesis testing, the test statistic (e.g. *F*, *t*, *r*) with confidence intervals, effect sizes, degrees of freedom and *P* value noted<br>*Give P values as exact values whenever suitable.*
☒	☐	For Bayesian analysis, information on the choice of priors and Markov chain Monte Carlo settings
☐	☒	For hierarchical and complex designs, identification of the appropriate level for tests and full reporting of outcomes
☐	☒	Estimates of effect sizes (e.g. Cohen's *d*, Pearson's *r*), indicating how they were calculated

Our web collection on statistics for biologists contains articles on many of the points above.

Software and code

Policy information about availability of computer code

Data collection	The tool (Clinical Data Analysis and Reporting System) developed by Hong Kong Hospital Authority was used to retrieve Hong Kong data.<br>The tool (the National Health Insurance Research Database) developed by the Health and Welfare Data Center HWDC and SAS 9.4 were used to retrieve Taiwan data.<br>N/A to the Nordic and New Zealand site.
Data analysis	Custom codes were developed for Statistical Analysis System (SAS) v9.4 (SAS Institute, Cary, NC, USA) and R Foundation for Statistical Computing version 3.6.0 (Vienna, Austria) .

For manuscripts utilizing custom algorithms or software that are central to the research but not yet described in published literature, software must be made available to editors and reviewers. We strongly encourage code deposition in a community repository (e.g. GitHub). See the Nature Portfolio guidelines for submitting code & software for further information.

Data

Policy information about availability of data

All manuscripts must include a data availability statement. This statement should provide the following information, where applicable:

- Accession codes, unique identifiers, or web links for publicly available datasets
- A description of any restrictions on data availability
- For clinical datasets or third party data, please ensure that the statement adheres to our policy

The Hong Kong data utilised in this study will not be accessible to external parties as the data custodians have not given permission due to concerns regarding patient privacy protection. Requests can be submitted to hacpaaedr@ha.org.hk (the Central Panel on Administrative Assessment of External Data Requests, the Hospital Authority, Hong Kong). It should be noted that the processing time for such requests may vary as the provided data will be customised for the specific purpose of each project.
Individual level data from the Nordic countries were used under license for the current study and cannot be made publicly available due to data privacy laws. The data are available from the data custodians of the health registers after obtaining the necessary permissions in Finland, Iceland, Norway, and Sweden.
Due to data privacy laws, individual level data from the New Zealand cannot be make publicly available.
The National Health Research Insurance Database of Taiwan can only be accessed at the Health and Welfare Data Center (HWDC) due to data privacy concerns.

Research involving human participants, their data, or biological material

Policy information about studies with human participants or human data. See also policy information about sex, gender (identity/presentation), and sexual orientation and race, ethnicity and racism.

Reporting on sex and gender	subgroup analyses by biological sexes were performed
Reporting on race, ethnicity, or other socially relevant groupings	N/A
Population characteristics	We identified 3,619,717 mother-child pairs to be included in the analysis (Figure 2). Overall, 8.0% (N=30,396), 4.1% (N=21,326), 13.7% (N=107,898), and 6.6% (N=126,425) of children were born to mothers with diabetes in Hong Kong, New Zealand, Taiwan, and the Nordic countries respectively. The mean ages of these children at the end of observation ranged from 7.7 to 11.5, and the percentage of boys ranged from 51.2% to 52.6%.
Recruitment	This study consisted of children from all live births within the site-specific study period thus selection bias is minimal.
Ethics oversight	The study utilised healthcare data obtained from Hong Kong, New Zealand, Taiwan, and Nordic countries. These data encompassed various sources such as electronic health records, registers, and insurance records. Each participating site followed the relevant local ethics and regulatory frameworks for study approval, namely, Finland: The Finnish Institute for Health and Welfare (THL/1551/6.02.00/2018, THL/1673/5.05.00/2019) and The Social Insurance Institution of Finland (Kela 148/522/2018 and Kela 117/522/2019); Hong Kong: University of Hong Kong/ Hospital Authority Hong Kong West Cluster (UW20-051); Iceland: National Bioethics Committee (VSNb2018060017/03.01); Norway: The Norwegian Data Inspectorate (17/02068/Norwegian Data Inspectorate) and the Regional Committee for Medical and Health Research Ethics (2017/2546/REC South-East Norway); New Zealand: New Zealand Health and Disability Ethics Committee (13789); Sweden: Swedish Ethical Review Authority (Dnr 2015/1826-31/2, 2017/2238-32, 2018/1790-32, 2018/2211-32, 2022-04004-02); Taiwan: National Cheng Kung University Human Research Ethics Committee (110-453).

Note that full information on the approval of the study protocol must also be provided in the manuscript.

Field-specific reporting

Please select the one below that is the best fit for your research. If you are not sure, read the appropriate sections before making your selection.

☒ Life sciences ☐ Behavioural & social sciences ☐ Ecological, evolutionary & environmental sciences

For a reference copy of the document with all sections, see nature.com/documents/nr-reporting-summary-flat.pdf

Life sciences study design

All studies must disclose on these points even when the disclosure is negative.

Sample size	This study consisted of children from all live births within the site-specific study period. We identified 3,619,717 mother-child pairs to be included in the analysis.
Data exclusions	No data were excluded from the analyses.
Replication	We have one person cross-checked the analyses using another software (R) independently to ensure the accuracy of our results.

Randomization	This is an observational study, neither randomisation or group allocation was conducted. Instead, propensity score (PS) fine-stratification weighting was used to address the differences in baseline covariates.
Blinding	This is an observational study, neither blinding or group allocation was conducted. Instead, pseudonymised patient-level electronic health data was used.

Reporting for specific materials, systems and methods

We require information from authors about some types of materials, experimental systems and methods used in many studies. Here, indicate whether each material, system or method listed is relevant to your study. If you are not sure if a list item applies to your research, read the appropriate section before selecting a response.

Materials & experimental systems

n/a	Involved in the study
☒	☐ Antibodies
☒	☐ Eukaryotic cell lines
☒	☐ Palaeontology and archaeology
☒	☐ Animals and other organisms
☒	☐ Clinical data
☒	☐ Dual use research of concern
☒	☐ Plants

Methods

n/a	Involved in the study
☒	☐ ChIP-seq
☒	☐ Flow cytometry
☒	☐ MRI-based neuroimaging
